# Development of lipopolyplexes for gene delivery: A comparison of the effects of differing modes of targeting peptide display on the structure and transfection activities of lipopolyplexes

**DOI:** 10.1002/psc.3131

**Published:** 2018-10-16

**Authors:** Robin Bofinger, May Zaw‐Thin, Nicholas J. Mitchell, P. Stephen Patrick, Cassandra Stowe, Ana Gomez‐Ramirez, Helen C. Hailes, Tammy L. Kalber, Alethea B. Tabor

**Affiliations:** ^1^ Department of Chemistry University College London 20, Gordon Street London WC1H 0AJ UK; ^2^ UCL Centre for Advanced Biomedical Imaging, Division of Medicine University College London London WC1E 6DD UK

**Keywords:** gene delivery, lipopolyplex, liposome, targeting peptide

## Abstract

The design, synthesis and formulation of non‐viral gene delivery vectors is an area of renewed research interest. Amongst the most efficient non‐viral gene delivery systems are lipopolyplexes, in which cationic peptides are co‐formulated with plasmid DNA and lipids. One advantage of lipopolyplex vectors is that they have the potential to be targeted to specific cell types by attaching peptide targeting ligands on the surface, thus increasing both the transfection efficiency and selectivity for disease targets such as cancer cells. In this paper, we have investigated two different modes of displaying cell‐specific peptide targeting ligands at the surface of lipopolyplexes. Lipopolyplexes formulated with bimodal peptides, with both receptor binding and DNA condensing sequences, were compared with lipopolyplexes with the peptide targeting ligand directly conjugated to one of the lipids. Three EGFR targeting peptide sequences were studied, together with a range of lipid formulations and maleimide lipid structures. The biophysical properties of the lipopolyplexes and their transfection efficiencies in a basal‐like breast cancer cell line were investigated using plasmid DNA bearing genes for the expression of firefly luciferase and green fluorescent protein. Fluorescence quenching experiments were also used to probe the macromolecular organisation of the peptide and pDNA components of the lipopolyplexes. We demonstrated that both approaches to lipopolyplex targeting give reasonable transfection efficiencies, and the transfection efficiency of each lipopolyplex formulation is highly dependent on the sequence of the targeting peptide. To achieve maximum therapeutic efficiency, different peptide targeting sequences and lipopolyplex architectures should be investigated for each target cell type.

## 1 INTRODUCTION

The delivery of oligonucleotide or genetic material to specific cells has been a long‐term goal for treatment of intractable diseases such as cancer, cystic fibrosis, retinal disorders, and cardiovascular disease. A range of potential gene delivery systems, both viral and non‐viral, have been used for the delivery of pDNA, siRNA, mRNA, and miRNA.^1^, ^2^ Viral gene delivery systems have high efficiencies and generally show good transfection properties in vitro and in vivo, with several in clinical trials, and one (Glybera) recently approved in the EU.^3^ However, in recent years, concerns about the potential safety of such approaches have led to a renewed interest in non‐viral gene delivery vectors, as these have lower immunogenicity, have the potential to deliver large payloads, and can be functionalised to target specific cell types. A range of nanoparticle‐based systems have been developed for gene delivery,^2^ with the most common vectors being those based on cationic lipids (lipoplexes), cationic polymers (polyplexes), or a combination of cationic lipids and cationic polymers (lipopolyplexes).^1^

Despite the advantages of non‐viral gene delivery vectors, in the past they have been slow to progress to clinical use due to their generally lower efficiency of gene delivery. Recent understanding of the barriers to efficient non‐viral vector delivery, such as nanoparticle instability in vivo, poor targeting to specific cells, and inefficient transport through biological barriers such as the cell membrane, has led to an increased number of candidate vectors currently in clinical trials.^2^ However, further improvements in these areas are still needed to realise the potential of gene‐based therapies, in particular in the treatment of cancers, where approaches such as suicide gene therapy,^4^ regulation of gene expression by delivery of miRNA,^5^ p53 replacement gene therapy,^6^ and redirection of T‐cell specificity towards cancer cells^7^ have recently shown promise. Targeting of nanoparticles to tumors can be passive or active. Nanoparticles of 100 to 200 nm in diameter tend to accumulate in tumours, through a combination of leaky tumor endothelium and ineffective lymphatic drainage, a phenomenon known as the enhanced permeability and retention effect (EPR).^8^ Whilst this passive targeting to tumors is undoubtedly important, inter‐ and intra‐tumoral heterogeneity of the tumor microenvironment means that the EPR effect may be more pronounced in some tumors than others.^9^ Moreover, it is also clear that accumulation in tumors is necessary, but accumulation on its own is not sufficient for cellular uptake.^10^ This frequently needs to be enhanced by the presence of cell‐specific targeting ligands on the surface of the nanoparticles. Such targeting ligands both enhance the selectivity of nanoparticles for cancer cells and may also trigger internalisation via mechanisms such as receptor‐mediated endocytosis. For example, exploiting the fact that epidermal growth factor receptor (EGFR) is over‐expressed on the surface of many cancer cell types, such as basal‐like breast cancer cells, has been very effective in targeting nanoparticles to such tumors.^11^ A range of preclinical studies have now demonstrated that active targeting improves the efficacy of nanoparticle‐based therapies for several cancer types,^12^, ^13^ and several targeted nanoparticle therapies are now in clinical trials.^13^ In addition, liposome‐based delivery systems and other nanoparticles that are shielded from the reticuloendothelial system with a surface coating of poly (ethyleneglycol) (PEG) or n‐ethylene glycol (n‐EG) have a significantly longer half‐life in vivo, allowing more of the nanoparticles to localise to the tumor.^14^

Lipopolyplex gene delivery systems combine the desirable features of lipoplexes and polyplexes with high in vivo transfection efficiencies and a nanoscale size (100‐200 nm). They are self‐assembling nanoparticles which can be formulated from a wide range of components, enabling them to be tailored to many different applications and have multiple functionalities (reviewed in Rezaee et al^15^). For example: formulation of LPD nanoparticles using cationic lipids and peptide sequences derived from protamine or histone resulted in enhancement of cell transfection in vitro^16^; in early work, RGD‐targeted LPD revealed a 30‐fold increase in cell transfection compared with the use of naked DNA^17^; lipopolyplexes incorporating a fusion protein consisting of the carboxy‐terminal domain of histone H1 and a nuclear localization signal gave transfection efficiencies up to 20‐fold higher than lipofectin/DNA complexes.^18^ We have previously developed targeted, environmentally responsive lipid:peptide:DNA (LPD) lipopolyplexes for gene delivery. These lipopolyplex formulations contain a bimodal peptide with a cationic sequence to bind and condense pDNA,^19^ a linker sequence (RVRR) which can be cleaved by enzymes within the endosome, and a targeting sequence.^20^ The formulation of the lipopolyplexes also includes cationic lipids such as DOTMA, and the helper lipid 1,2‐dioleoyl‐*sn*‐glycero‐3‐phosphoethanolamine (DOPE). The latter is believed to mediate release of the nanoparticle components from the endosome by fusion to the endosomal membrane and perturbing the structure to a non‐lamellar H_II_ phase.^21^ In the original paper describing the LPD vector^22^ and in more recent work^23^ the order of mixing of the lipid, peptide, and plasmid DNA were studied in detail, and it was shown that this order of mixing was crucial to ensure high transfection efficiencies. We have previously^24^ used a combination of FCS, freeze‐fracture electron microscopy, and fluorescence quenching experiments to prove the stoichiometry of the complex and demonstrate that the DNA is tightly condensed to the peptide in an inner core, which is surrounded by a disordered lipid layer, from which the integrin‐targeting sequence of the peptide partially protrudes, mediating internalization through receptor‐mediated endocytosis. Indeed, it has been shown by several groups (most recently Munye et al^25^) that cationic peptides more efficiently condense and package DNA than cationic liposomes, a phenomenon attributed to the higher charge density of the peptide molecules.^26^ We have recently developed lipopolyplexes which are sterically shielded by a shallow but even coverage of n‐EG conferred by incorporating novel cationic lipids with short n‐EG at the headgroup (*n* = 2‐6)^20^ and have studied the cellular uptake of the lipopolyplexes and the intracellular distribution of the components by confocal microscopy. We have also shown that liposomes formulated including these n‐EG lipids form nanoparticles that are shielded with a shallow, homogeneous n‐EG layer, and that these have much better cellular uptake than liposomes formulated with 1,2‐distearoyl‐sn‐glycero‐3‐phosphoethanolamine‐*N*‐[carboxy (polyethyleneglycol)_2000_] (DSPE‐PEG2000).^27^ We have recently used this approach to formulate lipopolyplexes that selectively transfected tumor cells with pDNA coding for a FRET biosensor and used this to monitor EGFR inhibition by tyrosine kinase inhibitors in vivo using quantitative FRET‐FLIM imaging.^28^ In this work, the bifunctional peptide incorporated peptide sequences targeting EGFR, conferring tumor selectivity and active targeting on these lipopolyplexes.

For these lipopolyplexes, the tumor selectivity and transfection efficiency both depend on how well the targeting moiety is displayed at the surface of the nanoparticle. A range of approaches for mounting the targeting moiety at the surface are possible^29^: as well as the bimodal peptide approach that we have adopted in previous work, other groups have successfully conjugated targeting peptides directly to the surface of liposomes,^30^ lipoplexes,^31^ and polyplexes.^32^ However, it is imperative to understand how subtle changes in the structures of the toolbox components can affect both the macromolecular architecture of the nanoparticles and also the selectivity, stability, and effective transfection in vivo of the resulting imaging probe.

In this paper, we have for the first time directly compared two different modes of attaching the targeting moiety to the lipopolyplex, via the bimodal peptide approach versus direct conjugation to the lipid. We have determined the effect that these two approaches have on the macromolecular structure of the lipopolyplex and its transfection efficiency. To develop this technology, we have used pDNA that has optical readouts through the expression of either firefly luciferase^33^, ^34^ or green fluorescent protein (GFP)^35^ with the aim that the lipopolyplex formulations could then be applied to other targeted gene‐based therapy approaches.

## 2 MATERIALS AND METHODS

### 2.1 General methods, reagents, and chemical synthesis

General methods for chemical synthesis are included in the Supporting Information. All chemicals were of commercial quality and have been used without additional purification. Unless otherwise stated chemicals were bought from Sigma Aldrich Co. Ltd. 1,2‐Dioleoyl‐*sn*‐glycero‐3‐phosphoethanolamine (DOPE) was purchased from Avanti Polar Lipids Inc. 1,2‐Di‐*O*‐octadecenyl‐3‐trimethylammonium propane (DOTMA) was synthesized according to literature procedures.^36^ 2,3‐Di‐((9*Z*)‐octadecenyloxy)propyl‐*N*‐(2‐{2‐[2‐(2‐hydroxyethoxy)ethoxy]ethoxy}ethyl)‐*N*,*N*‐dimethylammonium bromide (DODEG‐4) was synthesised as previously described.^37^ Experimental procedures for the synthesis, purification, and characterisation of the novel maleimide lipids DiOleyl‐Dimethyl‐Spacer‐Maleimide (DODSM) **1** and DiOleyl‐Spacer‐EthyleneGlycol3‐Maleimide (DOSEG3M) **2** are described in the Supporting Information. All peptides (Table S1) were synthesized via solid‐phase peptide synthesis using Fmoc chemistry. The synthetic procedures, purification methods, and compound characterisations are reported in the Supporting Information.

### 2.2 Plasmid DNA

The lentiviral transfer vector plasmid pSEW^38^ was engineered for the transient expression of firefly luciferase (5x FLuc)^34^ for bioluminescence, along with the enhanced GFP (eGFP) as a marker for fluorescence‐activated cell sorting (FACS) analysis.^35^ Plasmid DNA was amplified in bacteria (One Shot Top10 competent cells, Invitrogen) grown overnight in LB Broth with 100 μg/mL ampicillin, following heat‐shock transfection with the plasmid DNA. DNA was extracted and purified using a Qiagen Plasmid Maxi kit, according to the manufacturer's instructions, and eluted in de‐ionised water. DNA concentration and purity were measured using a spectrophotometer (NanoDrop 2000, Thermofisher), and the DNA stock was stored at −20°C until use.

### 2.3 Lipopolyplex formulation

*Bimodal peptide targeted lipopolyplexes:* For lipopolyplexes bearing their EGFR targeting peptide on the oligo‐lysine peptide, a mixture of 40 mol% DODEG‐4, 20 mol% DOTMA, and 40 mol% DOPE (formulation **F7**, Table 1) was prepared at a concentration of 1 mM in chloroform. The lipid mixture was slowly evaporated under reduced pressure to form a lipid thin film and further dried under high vacuum for at least 2 hours to ensure the complete removal of organic solvents. The thin film was then hydrated with deionised water to give a liposome solution with a final concentration of 1 mM lipids in water and sonicated in a VWR ultrasonic bath (45 kHz, effective power 80 W) to give an average size of around 200 nm. Subsequently, liposomes were diluted to a concentration of 100 μM and a solution of the corresponding bimodal peptide **P1** (K_16_‐RVRR‐YHWYGYTPQNVI), **P2 (**K_16_‐RVRR‐LARLLT), or **P3** (K_16_‐RVRR‐AEYLR) was added to a concentration of 5 μM, mixed and left standing for 5 minutes. Plasmid DNA was added to give a concentration of 1 μg/100 μL DNA per liposome‐peptide mixture to give bimodal peptide targeted lipopolyplexes.

**Table 1:** Molecular composition of lipids used for liposomal formulations **F1** to **F9** for the preparation of surface targeted and bimodal lipopolyplexes. Liposomes were made up to a total concentration of 1 mM lipid and subsequently diluted with de‐ionised water to give the appropriate concentrations for the preparation of lipopolyplexes (100 μM unless otherwise noted)

	Formulation	DODEG3 (Mol%)	DOPE (Mol%)	DOTMA (Mol%)	CHOL (Mol%)	DODSM (1) (Mol%)	DOSEG3M (2) (Mol%)
Surface targeted	F1	40	40	10	0	10	0
Surface targeted	F2	40	35	10	0	15	0
Surface targeted	F3	40	30	10	0	20	0
Surface targeted	F4	40	30	20	0	10	0
Surface targeted	F5	40	15	10	15	10	0
Surface targeted	F6	30	25	10	20	15	0
Bimodal	F7	40	40	20	0	0	0
Bimodal	F8	40	20	20	20	0	0
Surface targeted	F9	40	40	10	0	0	10

*Surface‐targeted lipopolyplexes:* For liposomes bearing the EGFR targeting peptide sequence on the lipid component, mixtures of DODEG‐4, DOTMA, DODSM **1** or DODEG3SM **2** and DOPE were prepared in the ratios shown in Table 1, at a concentration of 1 mM in chloroform. The lipid mixture was slowly evaporated under reduced pressure to form a lipid thin film and further dried under high vacuum for at least 2 hours to ensure complete removal of organic solvents. The thin film was then hydrated with deionised water to give liposome solutions **F1** to **F6** and **F9** (Table 1) with a final concentration of 1 mM lipids in water. These liposome solutions were then sonicated in a VWR ultrasonic bath (45 kHz, effective power 80 W) to give liposomes with an average size of approximately 200 nm. Peptide targeting sequences **P4** (CYHWYGYTPQNVI), **P5** (CLARLLT), and **P6** (CAEYLR), all with Cys residues at the *N*‐terminus, were then added to a final concentration of 1 mM and incubated for 2 hours. The liposomes were diluted to a lipid concentration of 500 μM and dialysed against deionised water (BioDesignDialysis Tubing (D001), 14000 MWCO) over 16 hours during which the water was changed 3 times. Subsequently, the peptide‐covered liposomes were diluted to a concentration of 100 μM, and a solution of oligo‐lysine (K_16_, **P7**) was added to a concentration of 5 μM, mixed and left standing for 5 minutes. Plasmid DNA was added to a concentration of 1 μg/100 μL DNA per liposome peptide mixture to give lipid bound targeted lipopolyplexes.

The final composition of all lipid formulations used for the preparation of both surface targeted and bimodal liposomes is summarised in Table 1.

### 2.4 Dynamic light scattering and zeta potential

The lipopolyplexes were characterised using dynamic light scattering and zeta potential measurements. Data were obtained using a Malvern Zetasizer Nano‐ZS (Malvern, UK). Aliquots of 10 μL were diluted to 500 μL in deionised water and analysed in triplicates. A representative sample of the prepared liposomal formulations and the resulting lipopolyplexes are reported in the Supporting Information (Tables S2, S3, and S4).

### 2.5 Cell culture

HCC1954 human breast cancer cells were grown in T175 flasks (Fisher Scientific, UK) in Roswell Park Memorial Institute (RPMI‐1640) medium (Invitrogen, UK), supplemented with 10% heat inactivated fetal calf serum (GIBCO, USA) in a humidified incubator at 37°C with 95% air and 5% CO_2_. Cells were grown to 80% confluence prior to trypsinisation, counting, and plating for in vitro experiments.

### 2.6 Liposome‐mediated transfection

HCC1954 cells were plated in six‐well plates (Corning, USA) at a concentration of 5 × 10^5^ cells per well in triplicate and incubated overnight to 80% confluency. Prior to adding liposomes, normal culture medium was aspirated from the wells, and the cells were washed once with phosphate buffered saline (GIBCO, USA), and 2 mL of serum free RPMI‐1640 was added. Then, 100 μL of either liposomes or phosphate buffered saline (control) was added to each well and incubated at 37°C. After 4 hours, 2 mL of normal culture medium was added and the mixture incubated for an additional 20 hours. After 24 hours, the incubation medium was aspirated from the wells and 2 mL of normal RPMI 1640 was added. At 24 and 48 hours after incubation, an in vitro bioluminescence assay (firefly luciferase expression) and FACS (eGFP expression) were performed as a read out of liposome mediated transfection efficiency.

Cell sorting was performed using a BD LSRFortessa (Becton Dickinson, USA) to assess the percentage of eGFP expression in each well. FACS analysis was performed using BD FACSDiva software (version 8.0.1).

The in vitro bioluminescence assay was performed using a IVIS Lumina (PerkinElmer, USA), and images were acquired immediately after adding D‐luciferin (300 μg/mL, beetle luciferin potassium salt, Promega, Madison, WI) using large binning and exposure time of 300 seconds. A region of interest was placed over each well, and the total radiance (photons/s) was quantified using Living Image software (version 4.5.2).

A two‐tailed paired t‐test assuming equal variances was performed to determine significant difference, at the 5% level statistical significance. Errors are given as standard deviation.

### 2.7 Fluorescence quenching

Fluorescence quenching experiments were carried out on an Agilent Cary Eclipse fluorometer equipped with a single water thermo cell holder. Spectral grade solvents were used for the fluorescence measurements. The preparation of fluorescein‐labelled pDNA and BODIPY‐labelled peptides **P8** and **P9** is described in the Supporting Information. Lipopolyplexes for fluorescence measurements were prepared at 200‐μM lipid, 0.02 μg/μL plasmid DNA, and 10 mM peptide. The lipopolyplex samples were diluted to a lipid concentration of 30 μM, and a total volume of 1.4 mL before fluorescence spectra were recorded and acrylamide was added.^24^ Collisional quenching of the fluorescence was plotted using the Stern‐Volmer equation:

$$\frac{F_{0}}{F}=k_{Q}\;\tau_{0}\;\left[Q\right]$$

where F_0_ and F are the fluorescence intensities in the absence and presence of quencher Q, respectively, k_Q_ is the bimolecular quenching constant, and τ_0_ is the lifetime of the fluorophore in the absence of quencher. For species in which a single population of fluorophores is present, all equally accessible to the quenching agent, a plot of F_0_/F will give a linear graph.^39^

## 3 RESULTS AND DISCUSSION

### 3.1 Design of lipopolyplexes: peptide targeting and lipopolyplex architecture

In order to exploit the overexpression of the EGF receptor at the surface of the basal‐like breast cancer cells used in this study, three peptides were investigated that have recently been reported to target this receptor. The GE11 (YHWYGYTPQNVI),^40^ D4 (LARLLT),^41^ and AE (AEYLR)^42^ peptide sequences were previously identified as suitable ligands, via phage display or in silico peptide library screening. These sequences have been successfully used to target liposomes^30^, ^41^ and polyplexes^42^ to tumor cells. As previous studies have shown that the transfection efficiency and route of cellular uptake of such targeted nanoparticles were highly dependent on cell type,^43^ we elected to compare all three of these sequences in the lipopolyplex formulations.

**Figure 1:**
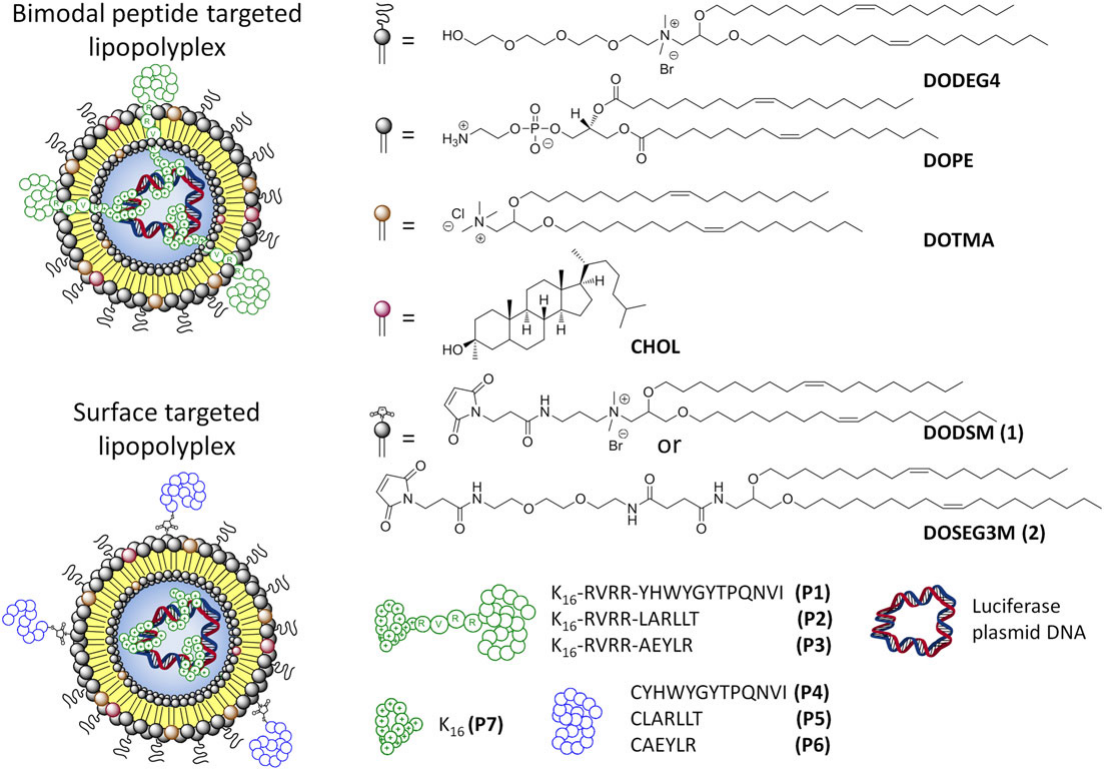
Schematic representation of (top) bimodal peptide targeted lipopolyplexes formulated with a bimodal peptide consisting of an EGFR targeting sequence, a furin cleavable RVRR linker, a DNA condensing K_16_ sequence, and (bottom) surface targeted lipopolyplexes formulated using an EGFR targeting sequence bioconjugated to the lipid bilayer, and a separate K_16_ peptide to condense the DNA. The lipid, peptide, and plasmid DNA components used in these experiments are shown on the right hand side

The lipopolyplex formulation utilised was based on a single, multifunctional peptide which will both condense pDNA and target the nanoparticle to cell surface receptors. We have previously shown that this necessitates the targeting peptide protruding through the surface of the lipid layer, resulting in the “bifunctional peptide” design shown in Figure 1.^24^ For these lipopolyplex formulations, peptides **P1**, **P2**, and **P3** were synthesised, with K_16_ condensing sequences, the RVRR enzymatically cleavable linker, and the GE11 (**P1**), D4 (**P2**), or AE (**P3**) sequences. However, it was reasoned that the targeting sequence might be more sterically accessible to the receptor if it was conjugated to the surface of the nanoparticle, with a separate K_16_ peptide (**P7**) included in the formulation to condense the pDNA, giving the “surface targeted” design (Figure 1).

Bioconjugation of targeting moieties to lipids or to liposomes is typically carried out via the reaction of thiol‐functionalised targeting moieties to malemide‐derivatised lipids, using click chemistry, or amide bond formation,^29^, ^44^ although hydrazone linkages^45^ and Staudiger ligations^44^ have also been reported. The majority of peptide‐targeted liposomes to date have relied on the post‐formulation bioconjugation of thiol‐functionalised targeting moieties to liposomes containing DSPE‐PEG2000‐Mal,^29^, ^30^, ^41^ although other maleimide‐containing lipids with shorter or no PEG linkages have been reported.^31^, ^46^, ^47^ For the surface targeted lipopolyplexes two lipids with maleimide moieties at their head group were synthesised, for incorporation into the liposome formulations and then conjugation to peptide targeting sequences terminating in Cys residues. DODSM (DiOleyl‐Dimethyl‐Spacer‐Maleimide) **1** was designed to be a cationic lipid analogue of DOTMA, whilst DOSEG3M (DiOleyl‐Spacer‐EthyleneGlycol3‐Malelimide) **2** is a neutral lipid with a short n‐EG spacer between the lipid headgroup and the maleimide moiety.

**Scheme 1:**
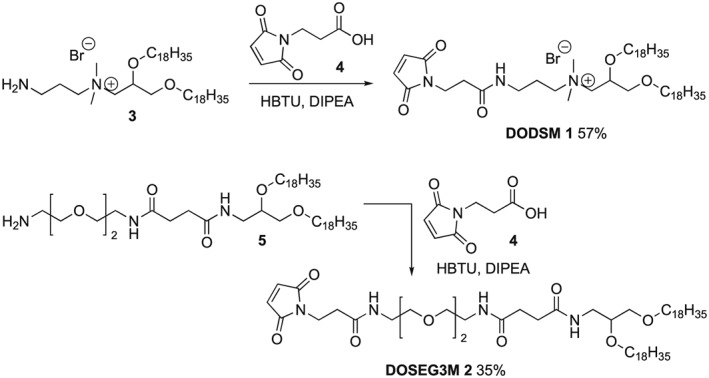
Synthesis of DiOleyl‐dimethyl‐spacer‐Maleimide (DODSM, **1**) and DiOleyl‐spacer‐EthyleneGlycol‐3‐Maleimide (DOSEG3M, **2**)

DODSM (**1**), with the cationic headgroup, was designed to evaluate whether the relative location of the malemide on the surface of the liposomal formulation would have an impact on transfection efficiency or lipopolyplex architecture. Due to the positive charge of the headgroup, the maleimide moiety might be in closer proximity with the aqueous exterior of the lipopolyplex. The synthesis for both maleimide bearing lipids is shown in Scheme 1. Amine **3** was prepared according to literature procedures^37^ and reacted with *N*‐maleoyl‐ß‐alanine (**4**) using HBTU and DIPEA to give DODSM **1**. Amine **5** was prepared as previously described^27^ and similarly reacted with **4** to yield the desired maleimide lipid DOSEG3M **2**.

### 3.2 Formulation of targeted lipopolyplexes

The bimodal peptide targeted lipopolyplexes were formulated using our previously published procedures.^24^ A mixture of lipids was first used to produce liposomes and give formulations **F7** or **F8** (Table 1). Addition of the bimodal peptides **P1**, **P2**, or **P3** to **F7** or **F8** was followed by luciferase plasmid DNA, giving the desired bimodal targeted lipopolyplexes (Table S2).

**Scheme 2:**
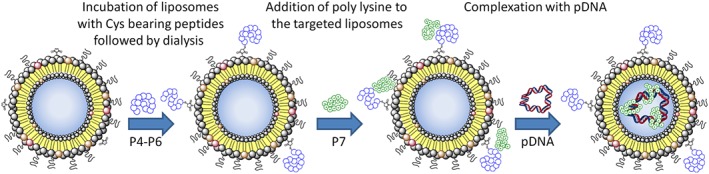
Schematic showing the preparation of surface targeted lipopolyplexes

Surface targeted lipopolyplexes for this study were prepared in a similar manner (Scheme 2). Liposomal formulations with either the charged lipid (**1**) (formulations **F1**, **F2**, **F3**, **F4**, **F5**, **F6**) or the neutral lipid (**2**) (formulation **F9**) incorporated into the lipid bilayer were prepared and subsequently incubated with Cys bearing targeting peptides **P4**, **P5**, or **P6** to give targeted liposomes. The excess unbound targeting peptide was then removed via dialysis and the liposomal formulation incubated with a DNA‐condensing peptide K_16_ (**P7**) followed by plasmid DNA to form the final surface targeted lipopolyplexes (Table S2). A schematic representation of the formation process is depicted in Scheme 2.

For both types of lipopolyplex, the initial liposome solution was sonicated to obtain an average particle size below 500 nm with a zeta potential of between +23 and +43 mV (Table S3). The liposomal formulation for bimodal targeted lipopolyplexes exhibited slightly higher surface charges than the surface targeted formulation despite using only 5 mol% more charged lipids in the bimodal formulation. The zeta potential difference is probably due to the shielding effect of the maleimide bearing lipids, analogous to the manner in which large PEG groups shield the charge of cationic lipoplexes.^48^ After the addition of the K_16_ containing peptides (**P1**‐**P3**, **P7**) and plasmid DNA, the average size of the samples slightly increased while the zeta potential slightly decreased to values ranging from +15 to +33 mV (Table S4) indicating the formation of stable lipopolyplexes with the negatively charged plasmid DNA reducing partially the overall positive charge. While it is known that lipoplexes of up to 250 nm are almost exclusively endocytosed by clathrin coated pits of non‐phagocytic B16 cells, particles of sizes around 500 nm are internalised via caveolae.^49^ No studies on the contribution of particle size on the internalisation pathway of lipopolyplexes has been conducted so far, but it can be assumed that the broad size distribution of the presented lipopolyplex with an average size above 250 nm may trigger different endocytosis mechanisms. However, a major contribution of receptor‐mediated endocytosis was expected due to the presence of EGFR targeting peptides.^20^

### 3.3 Transfection of HCC1954 human breast cancer cell line

**Figure 2:**
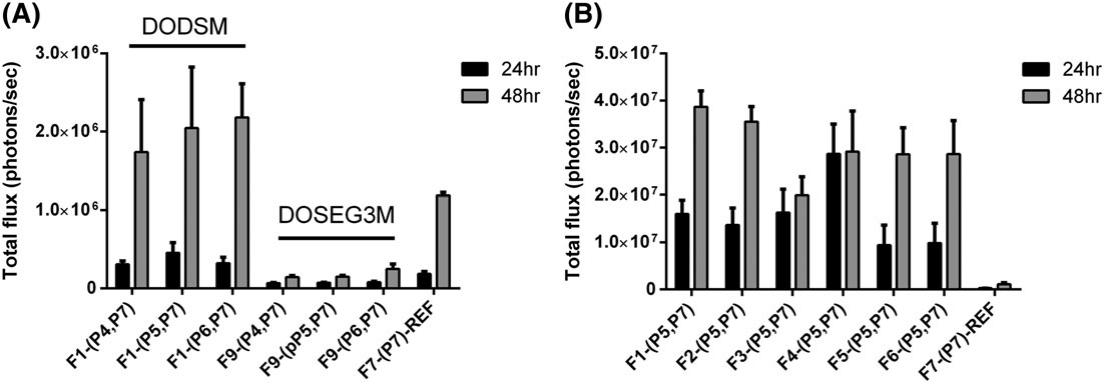
Optimisation of lipid formulations for the surface targeted lipopolyplexes. A, Total luciferase emission of HCC1954 cells transfected with lipopolyplexes prepared from lipid formulations **F1** (DODSM), **F9** (DOSEG3M), and **F7** (no maleimide‐lipid) covalently linked to EGFR targeting peptides (**P4‐P6**) via maleimide‐thiol crosslinking and complexed with K_16_ (**P7**) and luciferase pDNA. B, Total luciferase emission of HCC1954 cells transfected with lipopolyplexes prepared from liposomal formulations **F1‐F7** containing DODSM, covalently linked to EGFR targeting peptide **P5** and complexed with **P7** and luciferase pDNA. Error bars are standard deviation (*n* = 3)

We initially investigated the surface targeted lipopolyplexes, which were optimised by changing different parameters such as the lipid composition or the ratio between peptide and DNA. The relative merits of using either maleimide lipid **1** or **2** to conjugate the targeting peptides (**P4**‐**P6**) to the exterior of the liposome were first examined. As described above, formulation **F1** (with DODSM **1**) or formulation **F9** (with DODEG3SM **2**), respectively, were conjugated to **P4** to **P6**, followed by formulation into lipopolyplexes by the addition of 5 μM K_16_ peptide (**P7**) and then 0.01 μg/μL pDNA. As a control, formulation **F7** was complexed with peptide **P7** and pDNA to give the non‐targeted lipopolyplex **F7‐(P7)‐REF** (Table S2). The transfection efficiency of the plasmid DNA into HCC1954 cells was assessed via bioluminescent light emission in radiance (firefly luciferase expression) and FACS analysis (eGFP expression) after 24 and 48 hours, respectively (Figure 2A). When DODSM **1** was used as the maleimide bearing component (lipopolyplexes **F1‐(P4,P7)**, **F1‐(P5,P7)**, and **F1‐(P6,P7)**) the bioluminescence output was similar to the control non‐targeted lipopolyplex **F7‐(P7)‐REF** at 24 hours but exhibited a distinctive increase (1.5‐1.8 fold) at 48 hours. This indicated good accessibility to the targeting functionalities and enhanced transcription of the plasmid DNA (Figure 2A) in the presence of **1**. Conversely, all lipopolyplexes containing DOSEG3M **2** (**F9‐(P4,P7)**, **F9‐(P5,P7)**, and **F9‐(P6,P7)**) showed significantly lower bioluminescence compared with the non‐targeted reference at both the 24 hours (*P* ≤ 0.05) and 48 hours (*P* ≤ 0.001) time point. One possible explanation for this is that the more polar maleimide headgroup‐linker moiety in DODSM protrudes further out from the bilayer, whereas the less polar headgroup‐linker moiety in DOSEG3M could interact with the hydrophobic part of the liposome bilayers.^50^

Lipopolyplexes containing DODSM **1** were therefore selected for further study. As lipopolyplexes bearing the EGFR targeting LARLLT peptide **P5** at the surface appeared from these preliminary studies to have slightly superior transfection efficiencies, we used this peptide sequence in studies to further optimise the composition of the lipid bilayer (Figure 2B). The ratios of DODSM, DOPE, and DOTMA were varied as shown in Table 1 (liposome formulations **F1**, **F2**, **F3**, **F4**), and lipopolyplexes containing cholesterol (liposome formulations **F5**, **F6**) were also investigated. All of the resulting lipopolyplexes showed significantly increased bioluminescence (*P* ≤ 0.001) for both 24 and 48‐hour time points compared with the non‐targeted reference lipopolyplex **F7‐(P7)‐REF**. This suggests that altering the lipid composition did not significantly affect targeted transfection efficiency. Liposomes containing **1** in the lipid bilayer in the range of 10 to 15 mol% (**F1‐(P5,P7)**, **F2‐(P5,P7)**), had the highest bioluminescence (48 hours). Whereas, increasing the percentage further to 20 mol% (**F3‐(P5,P7))** appeared to hinder transfection resulting in a reduction in bioluminescence at 48 hours. Increasing the overall charge of the complex (**F4‐(P5,P7)**) by increasing the percentage of DOTMA resulted in a more rapid transfection efficiency, with the highest bioluminescence at 24 hours. However, this did not increase to the extent of other lipopolyplexes at 48 hours. Finally, adding cholesterol to the formulation (**F5‐(P5,P7),** (**F6‐(P5,P7)**) also significantly reduced bioluminescence at both time points when compared with **F1‐(P5,P7)** and **F2‐(P5,P7)** (*P* ≤ 0.05), suggesting that cholesterol may affect the transfection efficiency, perhaps by increasing the rigidity of the bilayer. In order to maximise the number of sites for conjugation of the targeting peptide to the surface, it was decided to carry out further optimisation of the peptide targeting sequence based on the **F2‐(P5,P7)** formulation.

**Figure 3:**
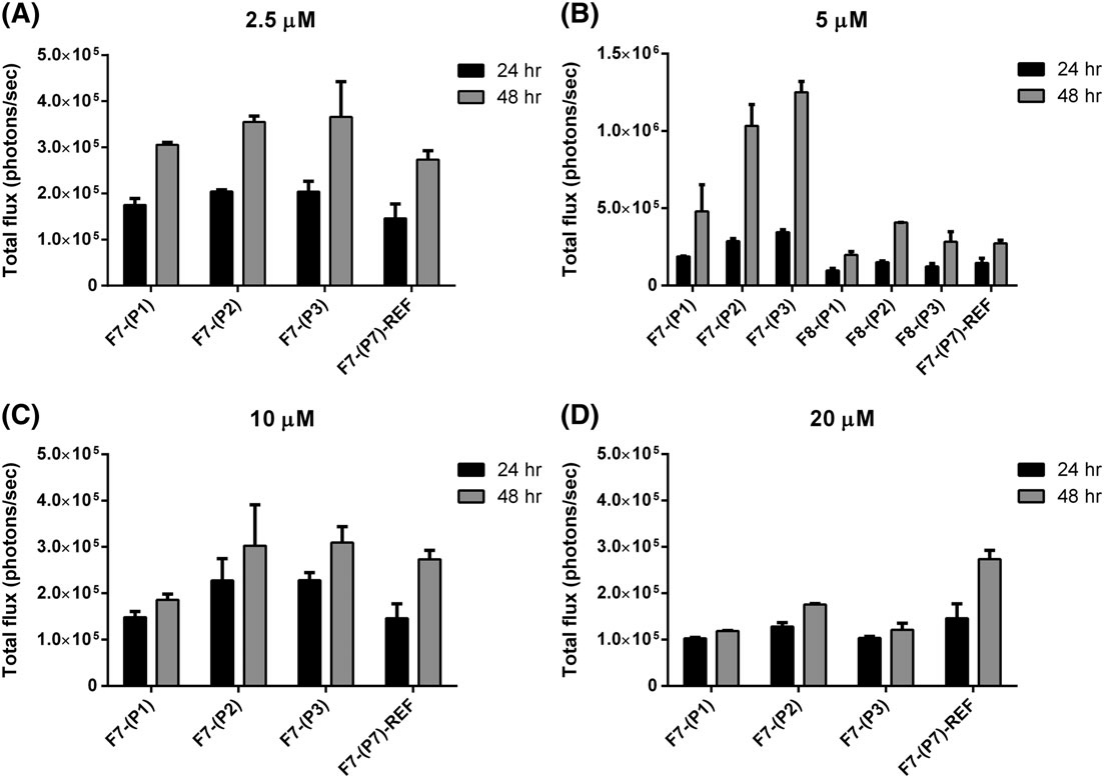
Optimisation of lipid formulations for the bimodal peptide targeted lipopolyplexes. (A‐D) Total luciferase emission of HCC1954 cells transfected with lipopolyplexes prepared from lipid formulation **F7** and **F8** (5 μM only—B) complexed with bimodal peptides (**P1**, **P2**, **P3**, and **P7‐REF**) at concentrations 2.5 μM (A), 5 (B), 10 μM (C), and 20 μM (D) and luciferase pDNA

Turning to the bimodal peptide targeted lipopolyplexes, peptides (**P1**, **P2**, and **P3**) were designed to have a pDNA binding sequence (K_16_) and a validated EGFR‐binding sequence (GE11 (YHWYGYTPQNVI),^40^ D4 (LARLLT),^41^ and AE (AEYLR)^42^) connected together by a furin‐cleavable linker sequence (RVRR), following our earlier work on the design of bimodal peptide targeted lipopolyplexes.^19^, ^20^, ^24^ Initially, the ratio between plasmid DNA and peptide was varied to find the optimized ratio that produces the highest bioluminescence emittance suggestive of the greatest luciferase expression (Figure 3A‐D). While keeping plasmid DNA concentration at 0.01 μg/μL, bimodal peptides were varied between 2.5 and 20 μM. As a control, the non‐targeted lipopolyplex formulation **F7‐(P7)‐REF** with a bimodal peptide concentration of 5 μM was used. A concentration of 5‐μM bimodal peptide was found to give the highest bioluminescence reading at both 24 and 48 hours for targeting peptides LARLLT (**F7‐(P2)**) and AEYLR (**F7‐(P3)**) compared with the control (**F7‐(P7)‐REF**) (*P* ≤ 0.05), whereas the longer GE11 (**F7‐(P1)**) targeting sequence showed only a slight enhancement of bioluminescence. Adding cholesterol to the lipopolyplex formulation at a concentration of 5 μM bimodal peptide (**F8‐(P1), F8‐(P2), F8‐(P3)**) or altering the concentration of bimodal peptide (**P1‐P3**) to either 2.5 or 10 μM had little effect on transfection efficiency, exhibiting similar levels of bioluminescence to the control (**F7‐(P7)‐REF**). However, increasing **P1** to **P3** to 20 μM appeared to significantly reduce gene expression levels compared with the non‐targeted reference at 48 hours (*P* ≤ 0.001).

**Figure 4:**
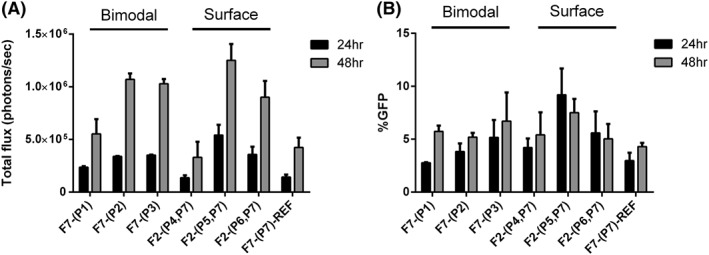
Comparison of luciferase emission in (A) HCC1954 cells and (B) HCC1954 cells expressing GFP (right) after incubation with the optimised formulations of bimodal peptide targeted (**F7‐(P1), F7‐(P2)**, **F7‐(P3)**) and surface targeted (**F2‐(P4,P7)**, **F2‐(P5,P7)**, **F2‐(P6,P7)**) lipopolyplex formulations

Having optimised the formulations for transfections using bimodal peptide targeted lipopolyplexes, we chose **F7** with the biomodal peptide concentration at 5 μM for the optimised bimodal peptide targeted lipopolyplex (Figure 3) and the **F2** formulation as the optimised surface targeted lipopolyplex (Figure 2). We then compared the two lipopolyplex targeting methods directly, using all three of the EGFR receptor targeting sequences (**P1‐P3** for the bimodal peptide and **(P4, P7)**‐**(P6, P7)** for the surface targeted). Again, these were compared with the control non‐targeted lipopolyplex **P7‐(P7)‐REF**. Bioluminescence was recorded as in previous experiments (Figure 4A) and compared with the expression of eGFP (Figure 4B) that was encoded on the same plasmid. FACS counting of eGFP expression was used to quantify the total percentage of transfected cells in a population. Bioluminescence was used to give a readout of transfection efficiency as the more luciferase produced results in a higher photon count when the same amount of luciferin is added. The percentage population of cells expressing eGFP was similar or higher for both the bimodal and surface targeted liposomes when compared with the non‐targeted reference **F7‐(P7)‐REF**. This suggests that the number of cells being transfected is similar if not better than the control. However, the LARLLT and AEYLR targeting sequences for both the bimodal peptide targeted lipopolyplexes (**F7‐(P2)** and **F7‐(P3)**) and surface targeted lipopolyplexes (**F2‐(P5, P7)** and **F2‐(P6, P7)**) had bioluminescence emission far higher than that of the non‐targeted reference **F7‐(P7)‐REF** at both 24 and 48 hours (*P* ≤ 0.01). As the percentage population by eGFP shows similar results to the non‐targeted reference, this suggests that the cells being transfected have a higher transfection efficiency which is most likely due to enhanced transcription of the luciferase gene. For the longer GE11 sequence, although the bimodal (**F7‐(P1)**) lipopolyplex had a higher bioluminescence photon count than the surface targeted (**F2‐(P4,P7)**) lipopolyplex, their expression was similar if not slightly lower (**F2‐(P4,P7)**) than the control (**F7‐(P7)‐REF**). We therefore undertook a structural investigation of the macromolecular architecture of the surface‐targeted and biomodal lipopolyplexes, in order to understand the observed differences in transfection efficiencies.

### 3.4 Characterisation of the macromolecular structure of the lipopolyplexes

**Figure 5:**
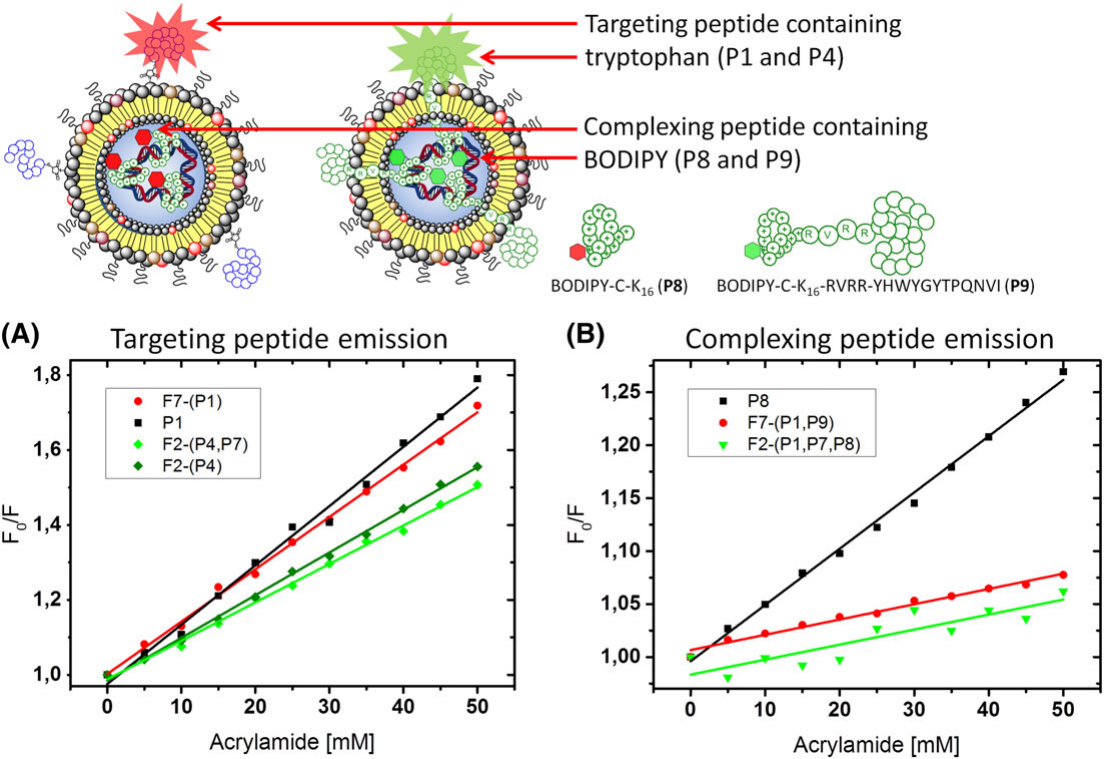
A, Stern‐Volmer plot for the quenching of bimodal lipopolyplex **F7‐(P1),** surface targeted lipopolyplex **F2‐(P4,P7)**, and surface targeted liposome **F2(P4)**. The overall lipid concentration = 200 μM, peptide = 10 μM, luciferase plasmid DNA 0.02 μg/μL). The GE11 EGFR targeting sequence (YHWYGYTPQNVI) was present in all peptides, and the Trp emission at λ_em_ = 340 nm (λ_exc_ = 280 nm) was quenched upon addition of acrylamide (0‐50 mM). B, Stern‐Volmer plot for the quenching of F4‐BODIPY in surface targeted lipopolyplex **F2‐(P4,P7)** incorporating a final concentration of 2‐μM F4‐BODIPY bearing peptide **P8**, bimodal lipopolyplex **F7‐(P1)** incorporating a final concentration of 2‐μM F4‐BODIPY bearing peptide **P9** (overall lipid concentration = 200 μM, peptide = 10 μM, F4‐BODIPY peptides = 2 μM, luciferase plasmid DNA 0.02 μg/μL). The BODIPY emission at λ_em_ = 530 nm (λ_exc_ = 557 nm) was quenched upon addition of acrylamide (0–50 mM)

In order to understand the differences in transfection efficiencies displayed by the bimodal and surface targeted lipopolyplexes, we conducted fluorescence quenching experiments to elucidate the location of the different components within the lipopolyplex.^24^ In order to study the accessibility and location of the targeting sequence itself, we used the GE11 (YHWYGYTPQNVI) sequence, as the intrinsic fluorescence of the Trp residue in this sequence made additional fluorophore labelling unnecessary. Thus, formulations **F7‐(P1)** (bimodal) and **F2‐(P4,P7)** (surface targeted) were made up at higher concentrations, and then increasing concentrations of acrylamide were added as a quencher.^39^ As a control, the quenching of the free peptide **P1** was taken to provide an indication of the quenching behaviour of a completely accessible peptide (Figure 5A). Furthermore, to provide a measure for a completely shielded fluorophore, 5(6)‐carboxyfluorescein was encapsulated into DPPC liposomes and subjected to the same quenching conditions used for the lipopolyplexes **(Figure** **S1** **)**. Quenching efficiencies for the Trp residue in the surface targeted lipopolyplex **F2‐(P4,P7)** were slightly lower than the quenching efficiency obtained from the free peptide **P1**. This may indicate that when the targeting peptide is mounted on the liposomal surface, it is sufficiently close to the lipid bilayer to limit the rotational freedom of the peptide and restrict access of the acrylamide quencher to the fluorophore. However, the bimodal formulation **F7‐(P1)** showed quenching efficiencies similar to the free peptide **P1**, indicating that in these lipopolyplexes the targeting part of the bimodal peptide protrudes through the lipid bilayer and is fully accessible to the acrylamide, as we have observed previously.^24^

In order to study the location of the DNA‐binding sequences in both the surface targeted and bimodal lipopolyplexes, we designed and synthesised two further peptides labelled with a fluorophore attached at the *N*‐terminal end of the DNA‐binding K_16_ sequence. Thus, the sequence CK_16_ was site‐selectively labelled with a fluorophore as follows. A perfluoro‐BODIPY^51^ functionalised in the para‐position of the perfluorophenyl ring was reacted via a nucleophilic aromatic substitution^52^ to give maleimido‐F4‐BODIPY. This was then conjugated to CK_16_ to give peptide **P8** (F4‐BODIPY‐CK_16_). Likewise, the peptide CK_16_‐RVRR‐YHWYGYTPQNVI was synthesised and site‐selectively conjugated to maleimido‐F4‐BODIPY via the Cys residue to give peptide **P9** (F4‐BODIPY‐CK_16_‐RVRR‐YHWYGYTPQNVI) (Figure 5). The bimodal lipopolyplex **F7‐(P1)** was then formulated incorporating a final concentration of 2 μM F4‐BODIPY bearing peptide **P9**, and the surface targeted lipopolyplex **F2‐(P4,P7)** was formulated incorporating a final concentration of 2 μM F4‐BODIPY bearing peptide **P8**. Similar quenching experiments using the free peptide **P8** as a control showed that the F4‐BODIPY labelled peptide is completely internalised in the bimodal lipopolyplex formulation **F7‐(P1,P9)** (Figure 5B) with the fluorophore protected from the acrylamide quencher. In the surface targeted lipopolyplex **F2‐(P4,P7,P8)**, good shielding from the quencher was equally observed, indicating internalisation of the BODIPY‐K_16_/DNA complex. Both linear Stern‐Volmer slopes resemble the one of completely encapsulated fluorescein (Figure S1).

In order to verify the location of the pDNA in the two lipopolyplexes, the luciferase plasmid DNA was labelled using a functionalised fluorescein (Supporting Information). However, no quenching of the lowest energy fluorescence emission band was recorded upon the addition of acrylamide (Supporting Information, Figure S2) for either the free labelled pDNA or the lipopolyplex encapsulated pDNA, indicating a shielding effect of the fluorophore by the more hydrophilic DNA. It is important to note that the overall emission of the labelled pDNA was generally low, resembling the spectra of a self‐quenched fluorophore, but after incorporation into a liposomal environment the typical fluorescence emission spectra of fluorescein recovered with a more then 4‐fold increase in emission intensity. Self‐quenching of fluorophores in macromolecules such as DNA is a known issue due to labelling of neighbouring sites supported via hydrophobic self‐assembly of the dye molecules.^53^ Despite that initial self‐quenching, it should be noted that once incorporated into the lipopolyplex the fluorescein labeled pDNA remains shielded from the quenching agent in the bulk water. The change in shape of the emission spectra can be attributed to the more hydrophobic environment of the lipopolyplex and the formation of a ternary complex with the cationic peptide which increases the distance between neighboring fluorophores.

## 4 CONCLUSIONS

Due to their demonstrated bio‐safety, reduced pathogenicity, low cost, and ease of production, lipopolyplexes remain a viable alternative to viral vectors for gene delivery applications. The addition of cell‐specific targeting ligands on the surface of liposome‐based nanoparticles enhances the cellular uptake and transfection efficiency. For instance, in a comparative study^54^ of the transfection efficiencies of lipopolyplexes formulated from bimodal peptides with and without targeting, lipopolyplexes with a scrambled targeting sequence showed 25% transfection, and lipopolyplexes with the targeting sequence removed showed 12% transfection, compared with lipopolyplexes with targeting sequence, indicating that the high transfection efficiency is at least partly a consequence of the targeting sequence. In another study,^41^ surface targeted liposomes bearing the GE sequence were compared with surface targeted liposomes with the sequence scrambled; the scrambled sequences had negligible binding to cells in vitro. However, a systematic investigation of the benefits of different methods of displaying the targeting peptide had not previously been reported.

In this study, we have for the first time compared two approaches to the design, synthesis, and formulation of cell‐surface receptor targeted lipopolyplexes for gene delivery. Surface‐targeted lipopolyplexes were prepared from liposomes to which targeting peptides had been attached, and bimodal peptides were formulated directly, using peptides which contained both DNA condensing and receptor targeting sequences. Three targeting sequences, previously validated to target the EGF receptor which is over‐expressed in many cancer cell lines, were investigated, together with a range of lipid formulations and maleimide lipid structures. The biophysical properties of the lipopolyplexes and their transfection efficiencies in a basal‐like breast cancer cell line were investigated, and fluorescence quenching experiments were used to probe the macromolecular organisation of the peptide and pDNA components of the lipopolyplexes.

Both approaches to lipopolyplex targeting gave reasonable transfection efficiencies, with little major differences between them. This reflects the observations from the fluorescence quenching experiments that in both types of liposomes the pDNA is condensed and shielded within the lipopolyplex, and also that in both cases the targeting moiety is accessible to some degree. Moreover, it is clear that the transfection efficiency of each lipopolyplex architecture is highly dependent on the sequence of the targeting peptide. The GE11 (YHWYGYTPQNVI) sequence gave the lowest transfection efficiency when incorporated in the surface targeted liposomes. In the fluorescence quenching experiments, the Trp residue of this sequence is partially shielded, suggesting that in this case the targeting sequence is partly shielded and thus is less accessible. However, the D4 (LARLLT) targeting sequence outperformed other surface targeted and bimodal lipopolyplexes, suggesting that this sequence has optimal accessibility to cell surface receptors.

In this work, we have demonstrated the versatility of differing approaches to functionalizing lipopolyplexes for targeted gene delivery which provides a toolbox for a more personalised targeting/treatment to specific cancer cell lines. Our findings indicate that there is little difference in transfection efficiency and targeting sequence display between the two approaches. Lipopolyplex targeting to cancer cell lines is clearly highly dependent on the targeting sequence used, and that selection and optimisation of both targeting sequence and lipopolyplex architecture should be carried out on a case‐by‐case basis, taking into account the cancer cell line of interest, the receptor binding of the targeting sequence, and the slightly greater synthetic complexity of the surface targeting approach.

## Supporting information




**Scheme S1:** Synthesis of Maleimido‐BODIPY (S3)
**Table S1** Peptide sequences used in lipopolyplex formulations.
**Table S2:** Summary of lipopolyplexes prepared for the transfection of HCC1954 cells. The names of the lipopolyplexes are made up as follows “Lipid formulation—(Peptides used)”.
**Table S3:** Biophysical characterisation of surface targeted liposomes **F2‐(P4)**, **F2‐(P5)**, **F2‐(P6)** and of untargeted liposome **F7**.
**Table S4:** Biophysical characterisation of lipopolyplexes after complexation with pDNA
**Figure S1:** Fluorescence quenching of free and liposomal fluorescein (5(6)‐Carboxyfluorescein 100 μM, DPPC 1 mM in HEPES 20 mM, pH 7.4, λ_exc_ = 466, λ_em_ = 516).
**Figure S2:** Fluorescein labeled pDNA emission in free solution and in lipopolyplex **F7‐(P1)**, overall lipid concentration = 200 μM, peptide = 10 μM, Luciferase plasmid DNA (0.02 μg/μL), λ_exc_ = 491 upon addition of 0 to 50 mM acrylamide.
