# Supplementary material for: Development of lipopolyplexes for gene delivery: A comparison of the effects of differing modes of targeting peptide display on the structure and transfection activities of lipopolyplexes
Source: J Pept Sci. 2018 Oct 16;24(12):e3131. doi: 10.1002/psc.3131 (PMC6282963; doi:10.1002/psc.3131)
Supplement: Supplementary file 1 — Scheme S1: Synthesis of Maleimido‐BODIPY (S3) Table S1 Peptide sequences used in lipopolyplex formulations. Table S2: Summary of lipopolyplexes prepared for the transfection of HCC1954 cells. The names of the lipopolyplexes are made up as follows “Lipid formulation—(Peptides used)”. Table S3: Biophysical characterisation of surface targeted liposomes F2‐(P4), F2‐(P5), F2‐(P6) and of untargeted liposome F7. Table S4: Biophysical characterisation of lipopolyplexes after complexation with pDNA Figure S1: Fluorescence quenching of free and liposomal fluorescein (5(6)‐Carboxyfluorescein 100 μM, DPPC 1 mM in HEPES 20 mM, pH 7.4, λexc = 466, λem = 516). Figure S2: Fluorescein labeled pDNA emission in free solution and in lipopolyplex F7‐(P1), overall lipid concentration = 200 μM, peptide = 10 μM, Luciferase plasmid DNA (0.02 μg/μL), λexc = 491 upon addition of 0 to 50 mM acrylamide. [file PSC-24-na-s001.docx]

**Development of lipopolyplexes for gene delivery: a comparison of the effects of differing modes of targeting peptide display on the structure and transfection activities of lipopolyplexes.**

Robin Bofinger, May Zaw-Thin, Nicholas J. Mitchell, P. Stephen Patrick, Cassandra Stowe, Ana Gomez-Ramirez, Helen C. Hailes, Tammy L. Kalber, and Alethea B. Tabor

**Supporting Information**

**Table of Contents**

| General methods | 2 |
| --- | --- |
| Synthesis of Maleimido-BODIPY (**S3**),  DODSM (**1**) and DOSEG3SM (**2**) | 3 |
| Solid phase peptide synthesis | 11 |
| Peptide sequences used in lipopolyplex formulations (Table S1) | 12 |
| Analytical data for peptides | 12 |
| Synthesis of BODIPY-labelled peptides | 16 |
| Analytical data for BODIPY-labelled peptides | 17 |
| Formulation of liposomes (Table S2) | 19 |
| Characterisation of liposomes and lipopolyplexes (Tables S3, S4) | 20 |
| Plasmid map | 21 |
| Labelling protocol for plasmid DNA  Fluorescence quenching data | 22  22 |
| References | 23 |

**General Methods:** Reagents for chemical synthesis were purchased from Sigma-Aldrich Co. Ltd. unless otherwise stated and used without further purification. All reagents were of commercial quality and used as received and all solvents anhydrous. Thin Layer Chromatography (TLC) was performed on aluminium backed Sigma-Aldrich TLC plates with F254 fluorescent indicator. Visualisation was performed by quenching of UV fluorescence or by staining the plates with potassium permanganate solution (1.5 g KMnO_4_, 10 g K_2_CO_3_, 1.25 mL 10% NaOH in 200 mL water), phosphomolybdic acid solution (10 % w/w in ethanol). Normal phase flash chromatography was carried out using silica gel (43–60 μm) supplied by Merck. Preparative HPLC was performed on a Varian ProStar HPLC system with a Model 210 solvent delivery module and a Model 320 UV detector. Analytical HPLC was performed on an Agilent 1260 Infinity HPLC with an Ace 5 C18 column 150 x 4.6 mm. Preparative purification was performed using a Varian column (100 x 21.2 mm, C18, 5 μm beads, flow rate of 10 mL/min) and analytical HPLC was performed using a Gemini column (250 x 4.6 mm, C18, 5 μm beads, flow rate of 1 mL/min). If not stated otherwise a solvent system of water (0.1% TFA) as solvent A and acetonitrile (0.1% TFA) as solvent B was used with a gradient of 5-40% B over 15 min followed by 40-95 B over 1 min and finally isocratic elution over 5 min at 95% B for preparative HPLC and a gradient of 5-95% B over 20 min used for analytical HPLC unless otherwise stated.

ESI-MS analysis was performed on a Waters Acquity Ultra Performance LC/MS system. ^1^H, ^19^F and ^13^C NMR spectra were recorded on Bruker Avance III 600 instrument at the field indicated. Chemical shifts (in ppm) were determined relative to tetramethylsilane (TMS) and referenced to residual protonated solvent. Coupling constants (*J*) were measured in Hertz (Hz), multiplicities for ^1^H coupling are shown as s (singlet), d (doublet), t (triplet), m (multiplet), or a combination of the above. Deuterated chloroform (CDCl_3_) was used as a solvent.

**Scheme S1:** Synthesis of Maleimido-BODIPY (S3)

The synthesis and characterisation of 4,4-difluoro-2,6-diethyl-1,3,5,7-dimethyl-8-perfluorophenyl-4-bora-3a,4a-diaza-s-indacene (perfluoro-BODIPY) has been reported elsewhere.^1^

**4,4-Difluoro-2,6-diethyl-1,3,5,7-dimethyl-8-(2-carboxyethylthio-2,3,5,6-tetrafluorophenyl)-4-bora-3a,4a-diaza-s-indacene (S2)**

Perfluoro-BODIPY **S1**^1^ (30 mg, 0.063 mmol) was dissolved in DMF (2 mL) and 3-mercaptopropionic acid (13 mg, 10.1 µL, 0.126 mmol) was added followed by TEA (26 µL, 0.189 mmol). The reaction mixture was monitored by TLC (CH_2_Cl_2_: formic acid 99:1). After 1 h, mercaptopropionic acid (20 mg, 0.19 mmol) was added and the reaction stirred for a further 1 h. The organic phase was diluted with CH_2_Cl_2_ and washed with sat. NaHCO_3_ solution (2 x 50 mL), brine (1 x 50 mL), dried (MgSO_4_) and evaporated and the crude product purified by silica column chromatography (CH_2_Cl_2_: formic acid, 99:1) to give **S2** (27 mg, 78%).^1^H NMR (600 MHz; CDCl_3_) δ 1.02 (t, *J* = 7.6 Hz, 6H), 1.53 (s, 6H), 2.33 (q, *J* = 7.6 Hz, 4H), 2.55 (s, 6H), 2.70 (t, *J* = 7.0 Hz, 2H), 3.28 (t, *J* = 7.0 Hz, 2H); ^19^F NMR (282 MHz; CDCl_3_) δ -132.1 (q, *J* = 12.5 Hz, 2F), -139.3 (q, *J* = 12.5 Hz, 2F), -145.5­ to -146.0 (m, 2 F); ^13^C NMR (150 MHz; CDCl_3_) δ 11.0, 12.9, 14.7, 17.2, 29.4, 33.9, 130.2, 133.9, 136.8, 156.1, 172.5; *m/z* (HRMS ES^+^) found [M+H 557.1879. C_26_H_28_BF_6_N_2_O_2_S requires 557.1869.

^1^H NMR

^19^F NMR

^13^C NMR

**4,4-Difluoro-2,6-diethyl-1,3,5,7-dimethyl-8-(2-(maleimido)ethyl 2,3,5,6-tetra-fluorophenylthio-propanoate-)-4-bora-3a,4a-diaza-s-indacene (S3)**

To acid **S2** (14 mg, 0.025 mmol) in DMF (1 mL), HBTU (70 mg, 0.18 mmol), then DIPEA (50 µL, 0.29 mmol) and *N*-(2-aminoethyl)maleimide trifluoroacetate salt (11 mg, 0.043 mmol) were added. The reaction mixture was stirred for 3 h then the solvent was evaporated under reduced pressure. The crude product was taken up in CH_2_Cl_2_ (20 ml) and washed with sat. NaHCO_3_ solution (2 x 50 mL), H_2_O (1 x 50 mL), dried (MgSO_4_) and evaporated. The crude product was purified by silica column chromatography (Petroleum ether 40-60:EtOAc, 30:70) to give **S3** (8 mg, 48%). ^1^H NMR (400 MHz; CDCl_3_) δ 1.02 (t, *J* = 7.6 Hz, 6H), 1.53 (s, 6 H), 2.34 (q, *J* = 7.6 Hz, 4H), 2.44 (t, *J* = 6.9 Hz, 2H), 2.54 (s, 6 H), 3.27 (t, *J* = 6.9 Hz, 2H), 3.46­–3.50 (m, *J* = 5.3 Hz, 2H), 3.69–3.75 (m, 2H), 5.96 (br s, 1H), 6.73 (s, 2H); ^19^F NMR (282 MHz; CDCl_3_) δ -132.3 (q, *J* = 12.5 Hz, 2F), -139.4 (q, *J* = 12.5 Hz, 2F), -145.5 to -145.60 (m, 2F); ^13^C NMR (150 MHz; CDCl_3_) δ 11.0, 12.9, 14.7, 17.2, 36.5, 37.5, 38.7, 39.5, 115.3 (t, *J* = 19.4 Hz), 116.1 (t, *J* = 19.9 Hz), 130.2, 133.9, 134.4, 136.9, 143.9 (dd, *J* = 250.7, 16.1 Hz), 147.4 (dd, *J* = 247.7, 14.3 Hz), 156.0, 170.3, 171.1; *m/z* (HRMS CI) found [MH]^+^ 678.2379 C_32_H_34_BF_6_N_4_O_3_S requires 678.2380.

^1^H NMR

^19^F NMR

^13^C NMR


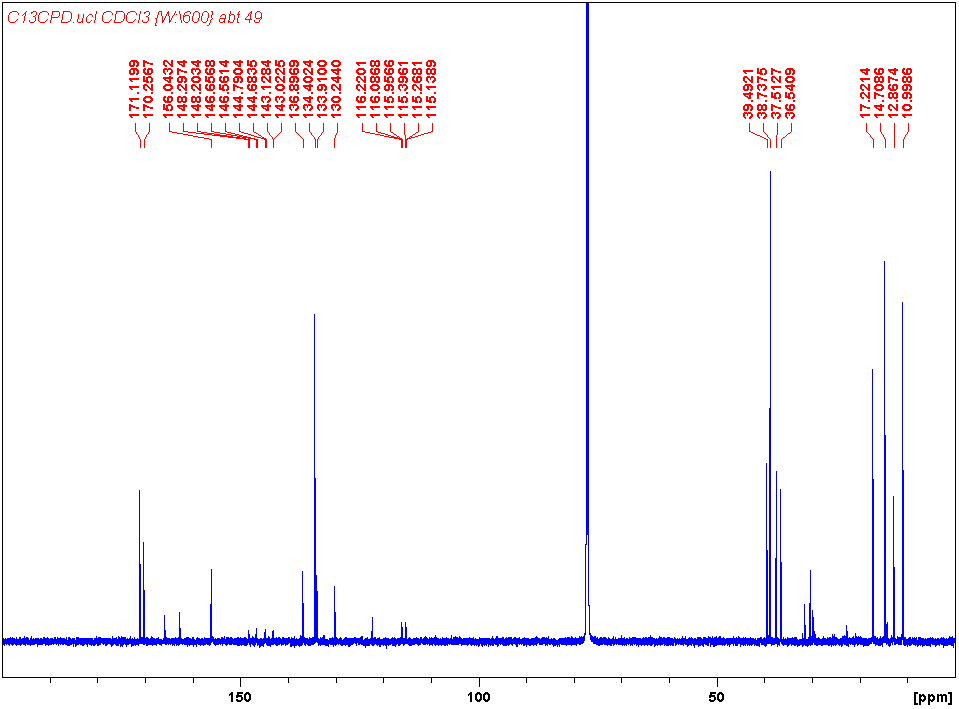


*N*-(3-Aminopropyl)-*N,N*-dimethyl-2,3-bis((*Z*)-octadec-9-enyloxy)propan-1-aminium bromide^2^ and *tert*-butyl (Z)-16-((Z)-octadec-9-enyloxy)-10,13-dioxo-3,6,18-trioxa-9,14-diazahexa­tria­cont-27-enyl carbamate^3^ were synthesized as previously reported.

***N*-(3-[3-{2,5-dioxo-2,5-dihydro-1*H*-pyrrol-1-yl}propanamido]propyl)-*N*,*N*-dimethyl-2,3-bis([(*Z*)-octadec-9-en-1-yl]oxy)propan-1-aminium bromide (DiOleyl-Dimethyl-Spacer-Maleimide) (DODSM, 1)**

The reaction was carried out under anhydrous conditions. To maleimidopropionic acid (150 mg, 0.887 mmol) in CH_2_Cl_2_ was added *N,N,N′,N′*-tetramethyl-*O-*(1*H*-benzotriazol-1-yl)uronium hexafluorophosphate (HBTU) (370 mg, 0.976 mmol) and diisopropylethylamine (DIPEA) (310 μL, 1.78 mmol). *N*-(3-Aminopropyl)-*N,N*-dimethyl-2,3-bis((*Z*)-octadec-9-enyloxy)propan-1-aminium bromide^2^ (652 mg, 0.860 mmol) in CH_2_Cl_2_ (2 mL) was then added and the reaction stirred at room temperature for 3 h. The solvent was removed under reduced pressure and the crude material purified via flash silica chromatography (2% MeOH in CH_2_Cl_2_) to give the titled compound as a colourless oil (443 mg, 57%). *v*max 2924, 2854, 1708 cm^–1^; ^1^H NMR (600 MHz; CDCl3) δ 0.86 (t, *J* = 7.0 Hz, 6H), 1.25-1.31 (m, 40H), 1.52–1.68 (m, 10H), 1.95–2.03 (m 8H)), 2.45 (t, *J* = 6.3 Hz, 2H), 3.13–3.23 (m, 1H), 3.18 (s, 3H), 3.19 (s, 3H), 3.32– 3.55 (m, 10H), 3.63–3.69 (m, 1H), 3.84 (dd, *J* = 13.5 and 7.2 Hz, 1H), 3.90–3.94 (m, 1H), 3.98­–4.05 (m, 1H) 5.34­–5-38 (m, 4H), 6.68 (s, 2H); ^13^C NMR (150 MHz; CDCl3) δ 14.3, 22.8, 26.1, 26.3, 27.3, 27.7, 29.1-29.9 (signals superimposed), 32.0, 32.7, 34.5, 35.0, 35.9, 39.2, 51.8, 53.0, 59.3, 64.6, 66.1, 68.4, 69.7, 72.2, 73.1, 129.9, 130.0, 134.4, 170.7, 170.9, 171.8; *m/z* (HRMS ES+) found [M]^+^ 828.7194. C_51_H_94_N_3_O_5_ requires 828.7193.

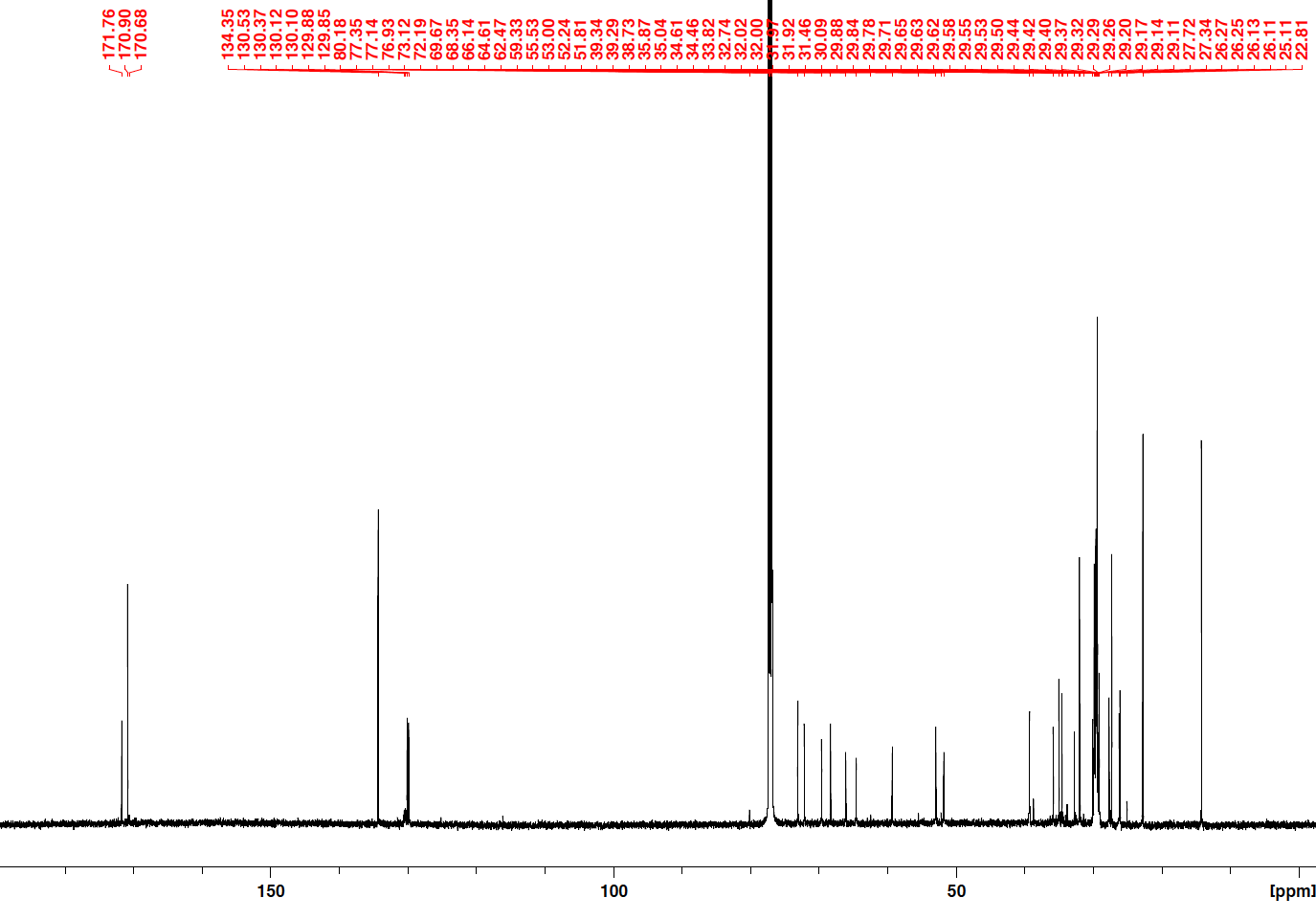


***N*^1^-(2-(2-(2-(Maleimido-ß-alaninamido)ethoxy)ethoxy)ethyl)-*N*^4^-(2,3-bis((*Z*)-octadec-9-enyloxy)propyl)succinamide (DiOleyl-Spacer-EthyleneGlycol3-Maleimide) (DOSEG3M) 2**

The reaction was carried out under anhydrous conditions. *tert*-Butyl (*Z*)-16-((*Z*)-octadec-9-enyloxy)-10,13-dioxo-3,6,18-trioxa-9,14-diazahexatriacont-27-enyl carbamate^3^ (112 mg, 0.122 mmol) was stirred in a mixture of CH_2_Cl_2_/TFA (1:1) for 3 h and the solvent was then removed *in vacuo*. To maleimidopropionic acid (25 mg, 0.15 mmol) in CH_2_Cl_2_ was added HBTU (69 mg, 0.18 mmol) and DIPEA (42 μL, 0.24 mmol). The deprotected amino-lipid (100 mg, 0.122 mmol) in CH_2_Cl_2_ was then added and the reaction was stirred at room temperature for 16 h. After this time the solvent was removed under reduced pressure and the crude material purified via flash silica chromatography (2% MeOH in CH_2_Cl_2_) to give the titled compound as a colourless oil (42 mg, 35%). *v*max 2922, 2853, 1707, 1645 cm^–1^; ^1^H NMR (600 MHz; CDCl3) δ 0.88 (t, *J* = 6.8 Hz, 6H), 1.20–1.35 (m, 44H), 1.50–1.65 (m, 4H), 1.95–2.01 (m, 8H), 2.51–2.55 (m, 6H), 3.24–3.28 (m, 1H), 3.37–3.63 (m, 18H), 3.84 (t, *J* = 7.2, 2H), 5.32–5.37 (m, 4H), 6.20 (br s, 1H), 6.53 (br s, 1H), 6.60 (br s, 1H), 6.69 (s, 2H); ^13^C NMR (150 MHz; CDCl3) δ 14.3, 22.8, 26.2, 27.3, 29.3–29.9 (signals superimposed), 30.2, 31.8, 31.9, 32.0, 32.7, 33.8, 34.5, 34.6, 39.4, 41.1, 69.85, 69.91, 70.3, 70.4, 71.5, 72.0, 76.8, 129.9, 130.1, 134.3, 170.0, 170.6, 172.2, 172.3; *m/z* (HRMS ES+) found [MH]^+^ 973.7568. C_56_H_101_N_4_O_9_ requires 973.7569.


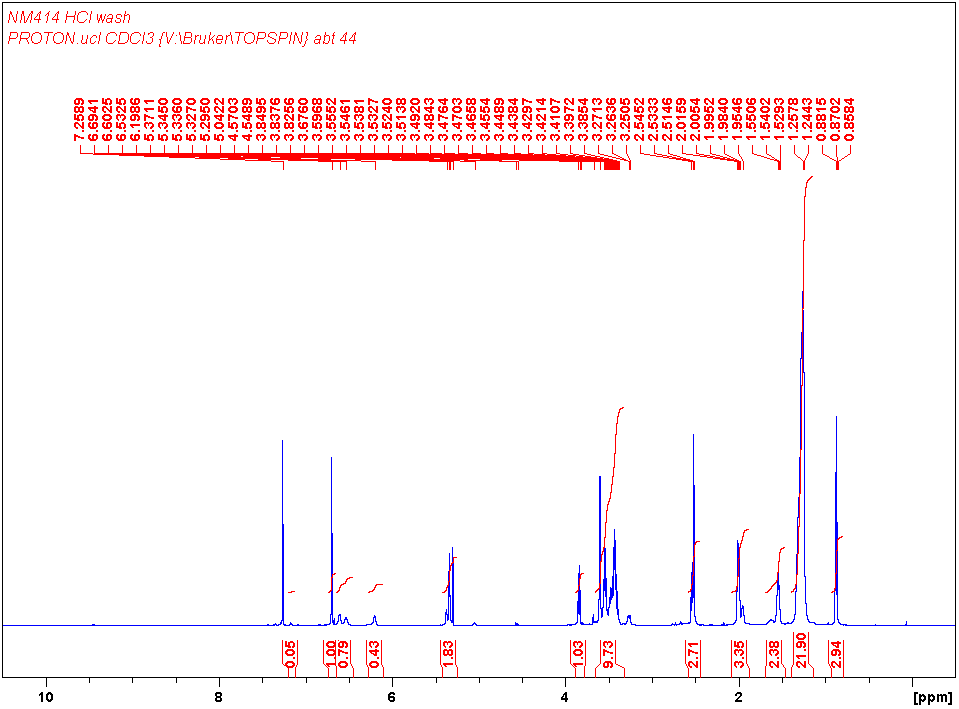


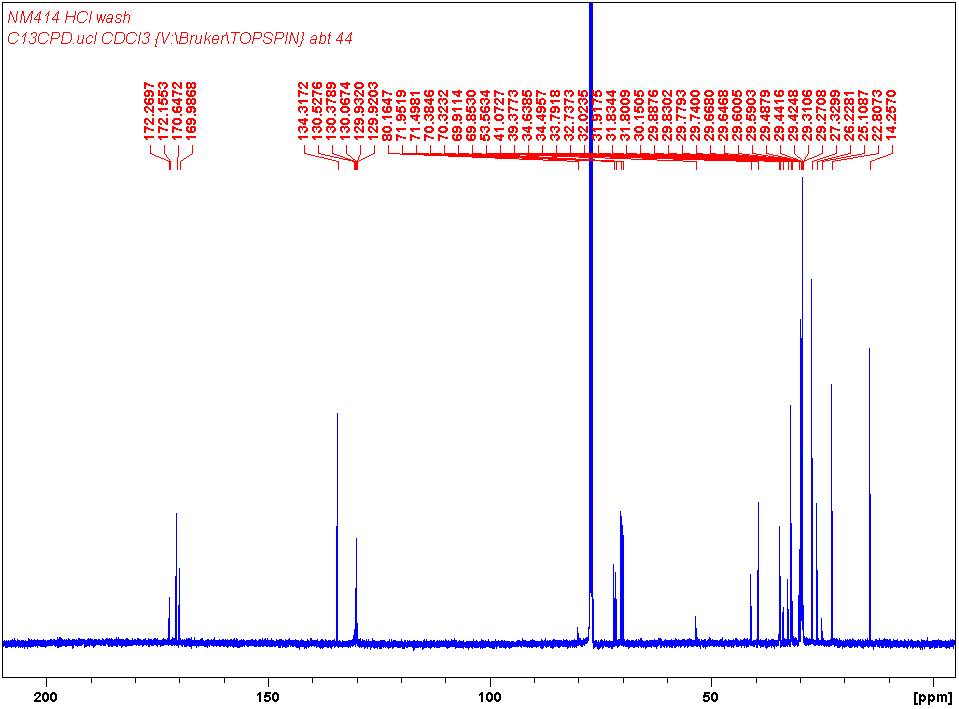


**Solid Phase Peptide synthesis**

**General methods** All peptides were synthesized using standard Fmoc solid-phase peptide synthesis on a MutiSynTech Syro I automated system. Pre-loaded TGT resin was used with standard HBTU/DIPEA coupling chemistry. All resins were pre-swelled in DMF for 10 min prior to the start of the synthesis. The total volume of all reagents in each step was 1.5 mL. *N*-Fmoc-protected amino acids were purchased from Novobiochem. Where required, the following side-chain protected amino acids were used: Fmoc-Lys(Boc)-OH; Fmoc-Arg(Pbf)-OH; Fmoc-Tyr(tBu)-OH; Fmoc-His(Trt)-OH; Fmoc-Thr(tBu)-OH.

*Fmoc deprotection:* Each deprotection step was allowed to proceed for 3 min with agitation for 20 s every minute in 40% piperidine in DMF at room temperature. The reagents were removed by filtration under vacuum and the resin washed with DMF (4 x 1.5 mL). The deprotection step was repeated using 20% piperidine in DMF with agitation for 20 s every minute for 10 min. The reagents were removed by filtration under vacuum and the resin washed with DMF (6 x 1.5 mL).

*Amino acid coupling:* To the resin was added 4 eq of the amino acid, 4 eq of HBTU and 8 eq of DIPEA to a total volume of 1.5 mL. The mixture was agitated for 20 s every 3 min for a total of 40 min at room temperature. The reagents were removed by filtration and the resin washed with DMF (4 x 1.5 mL).

*Peptide Cleavage:* After completion of the synthesis the peptide sequence was deprotected and cleaved from the resin by incubation in 2.5 mL of a cleavage mixture containing TFA/TES/EDT/H_2_O (9400:250:100:250 μL) for 3 h at room temperature. The cleavage cocktail was drained into a 15 mL falcon tube filled with diethyl ether and the peptide allowed to precipitate at -20 °C for 10 min. The suspension was centrifuged at 4000 rpm for 5 min, the supernatant was discarded and the pellet re-dissolved in diethyl ether. This purification process was repeated three times after which the precipitate was dissolved in 0.1% TFA containing HPLC grade water, frozen in liquid N_2_ and freeze-dried overnight. The peptides were purified using the HPLC system and columns specified in the general methods section. Fractions containing the correct peak were pooled, the solvent removed under reduced pressure to < 5 mL, and the solution freeze-dried overnight. The resulting crystalline powder was analyzed *via* HPLC and ESI-MS.

The purification and characterisation of peptides **P1**, **P2** and **P7** have been previously reported.^4^

| **Peptide** | **Sequence** |
| --- | --- |
| **P1** | K_16_-RVRR-YHWYGYTPQNVI |
| **P2** | K_16_-RVRR-LARLLT |
| **P3** | K_16_-RVRR-AEYLR |
| **P4** | CYHWYGYTPQNVI |
| **P5** | CLARLLT |
| **P6** | CAEYLR |
| **P7** | K_16_ |
| **P8** | (F4-BODIPY)-CK_16_ |
| **P9** | (F4-BODIPY)-CK_16_RVRRYHWYGYTPQNVI |

**Table S1** Peptide sequences used in lipopolyplex formulations.

**CYHWYGYTPQNVI (P4)**

Analytical HPLC trace of purified CYHWYGYTPQNVI; R_t_ 10.7 min (5-95% B over 20 min, λ = 220 nm).

**
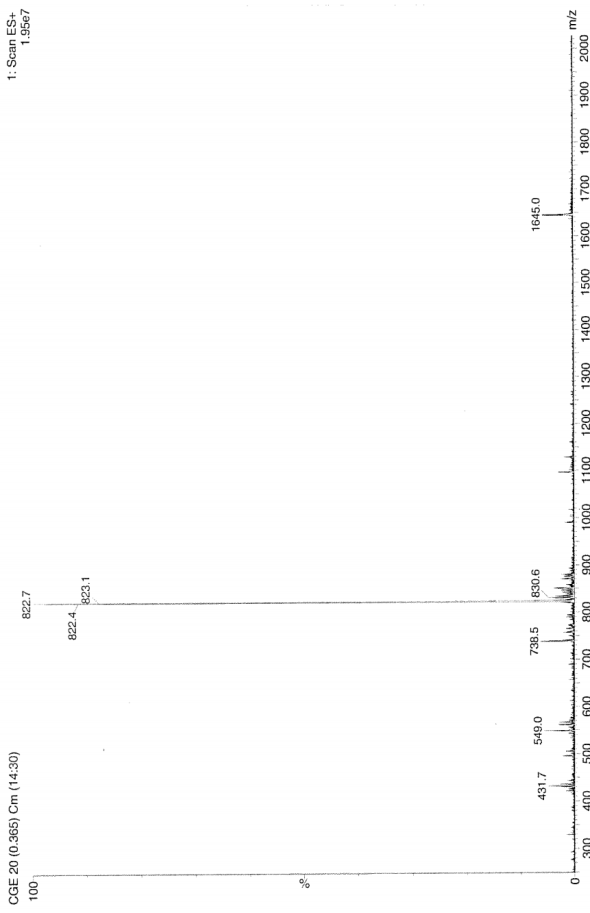
**

*m/z* Calculated Mass (ESI^+^): 1644.82 [M+H]^+^, 822.91 [M+2H]^2+^, 548.94 [M+3H]^3+^.Mass found: 1645.0 [M+H] ^+^  822.7 [M+2H]^2+^, 549.0 [M+3H]^3+^.

**CLARLLT (P5)**

Analytical HPLC trace of purified CLARLLT; R_t_ 9.5 min (5-95% B over 20 min, λ = 220 nm).

**
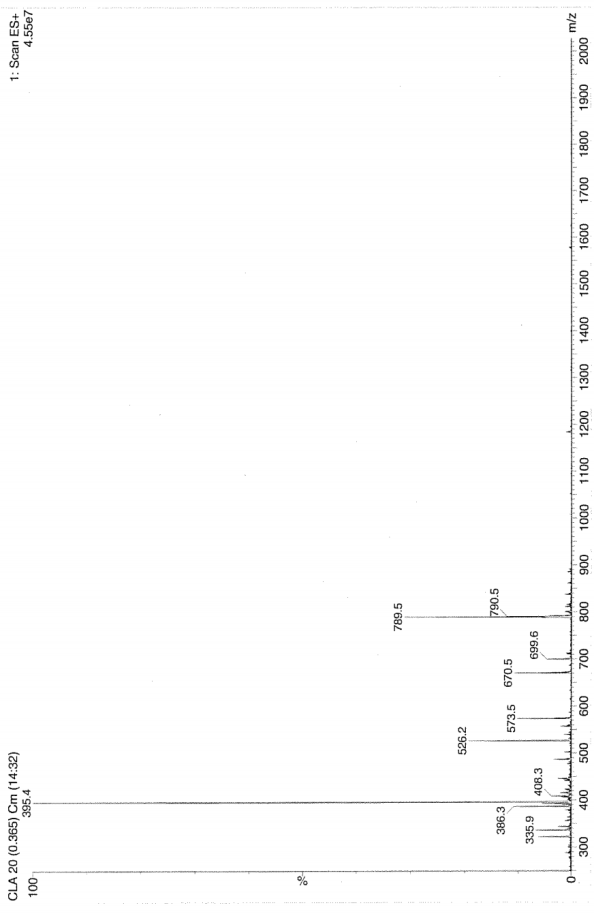
**

*m/z* Calculated Mass (ESI^+^): 789.47 [M+H]^+^, 395.23 [M+2H]^2+^.

Mass found: 789.5 [M+H] ^+^  395.4 [M+2H]^2+^.

**CAEYLR (P6)**

Analytical HPLC trace of purified CAEYLR; R_t_ 8.55 min (5-95% B over 20 min, λ = 220 nm).


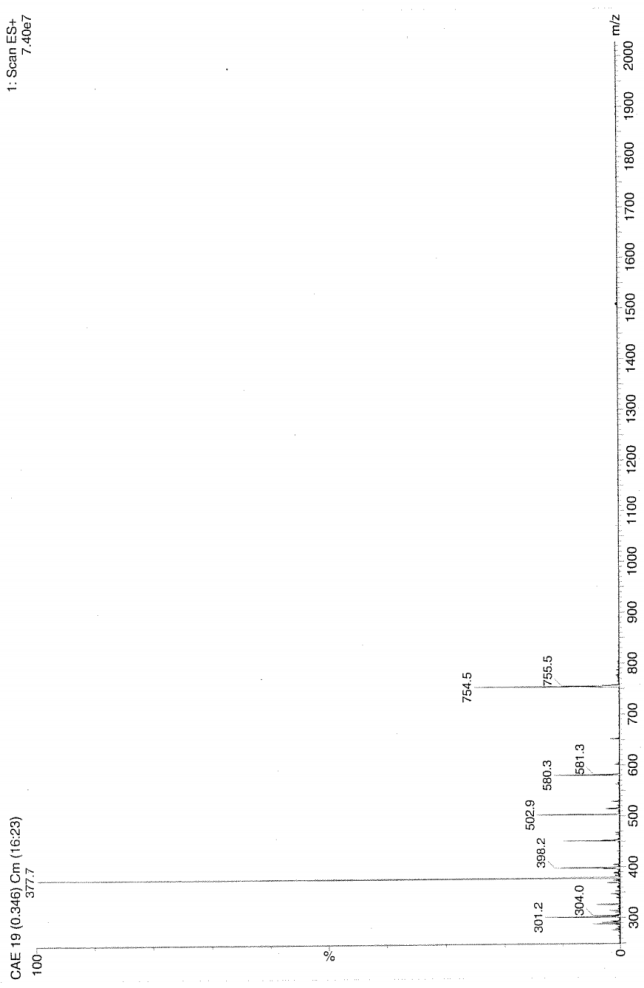


*m/z* Calculated Mass (ESI^+^): 754.36 [M+H]^+^, 377.68 [M+H]^2+^.

Mass found: 754.5 [M+H] ^+^  377.7 [M+2H]^2+^.

**K16-RVRR-AEYLR (P3)**

Analytical HPLC trace of purified K16-RVRR-AEYLR; R_t_ 7.29 min (5-95% B over 20 min, λ = 220 nm).

**
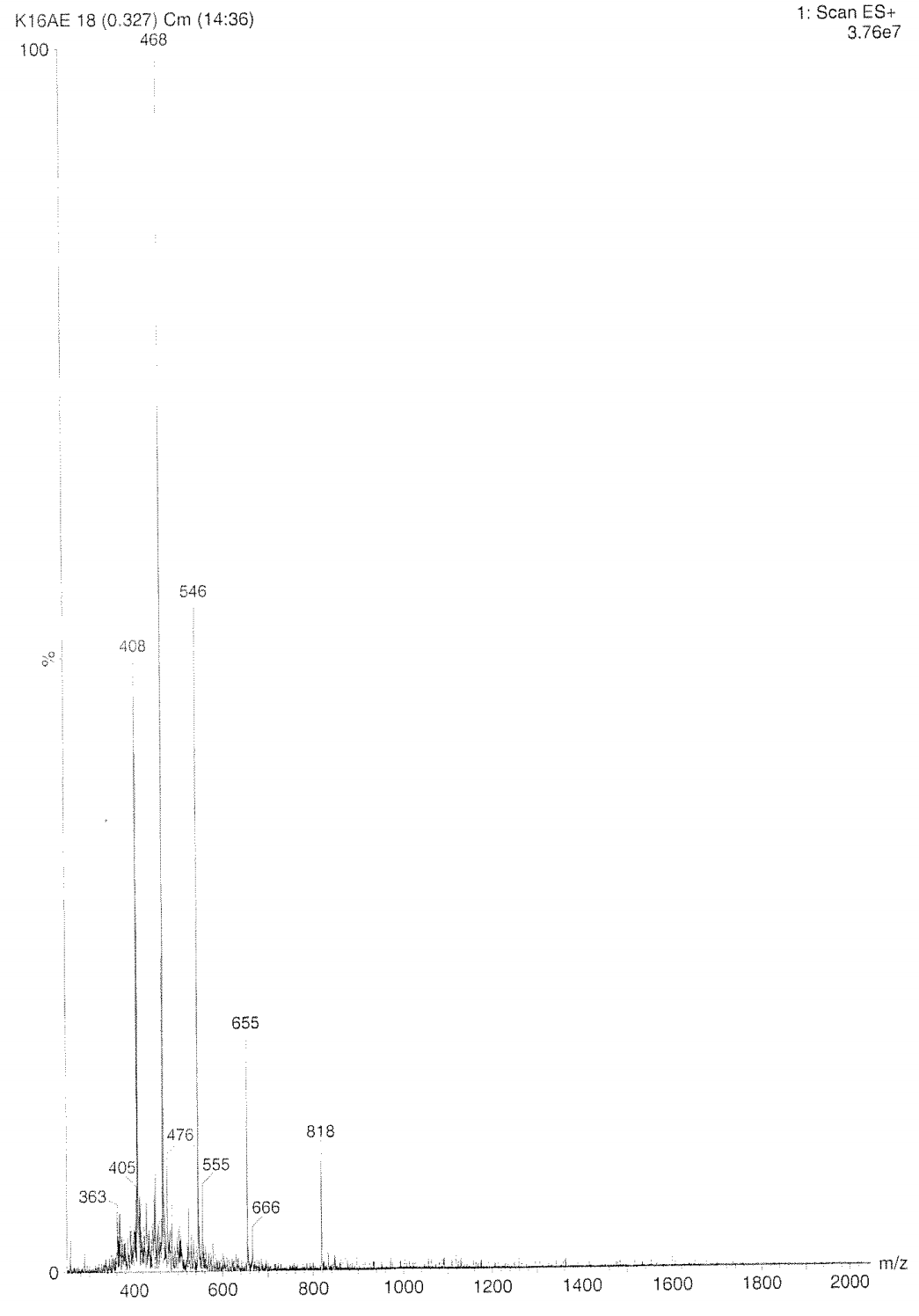
**

*m/z* Calculated Mass (ESI^+^): 3269.19 [M]^+^, 818.29 [M+4H]^4+^, 654.83 [M+5H]^5+^, 545.86 [M+6H]^6+^, 468.02 [M+7H]^7+^, 409.64 [M+8H]^8+^, 364.24 [M+9H]^9+^. Mass Found: 818 [M+4H]^4+^, 655 [M+5H]^5+^, 546 [M+6H]^6+^, 468 [M+7H]^7+^, 408 [M+8H]^8+^, 363 [M+9H]^9+^.

**K16C**

**
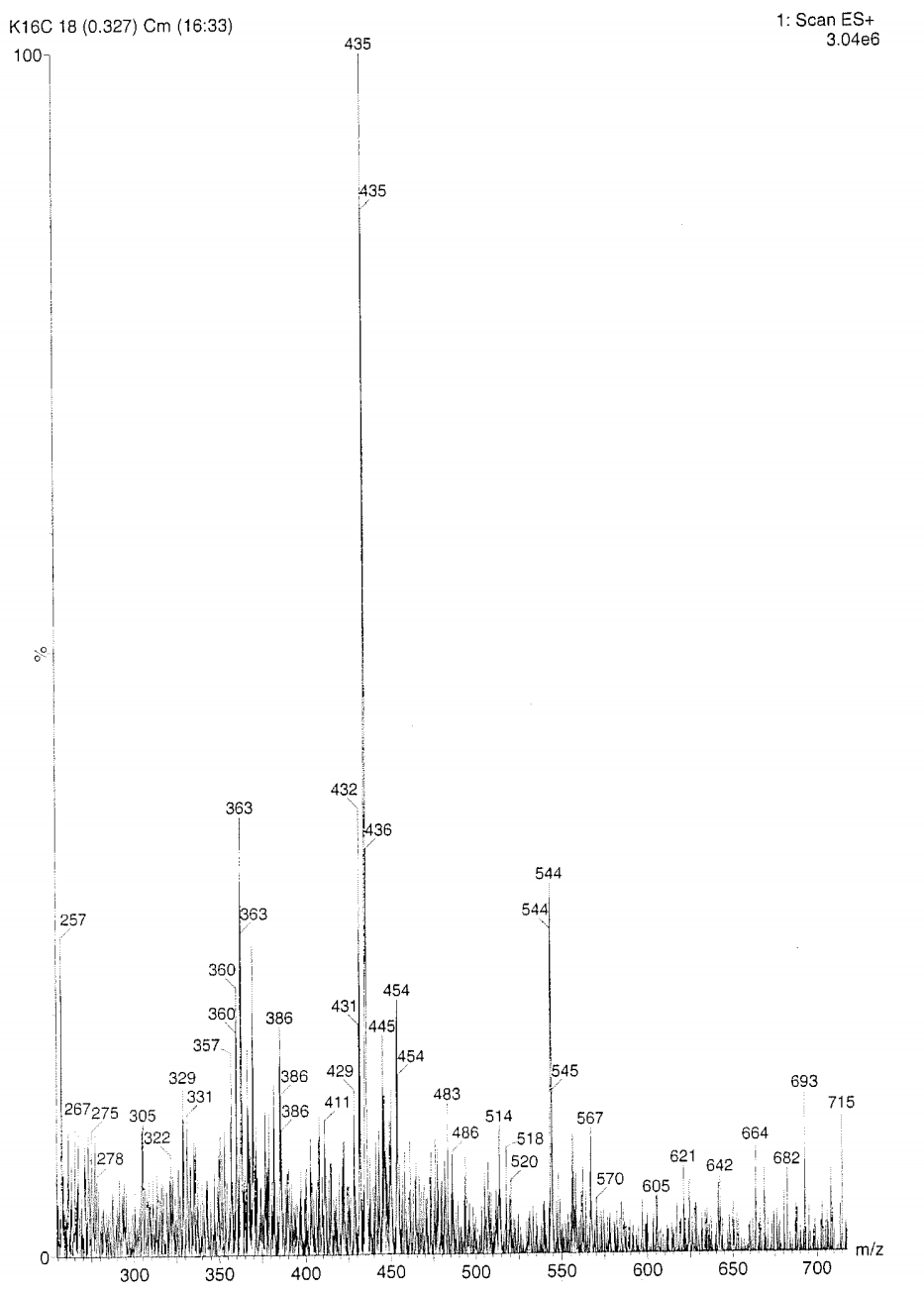
**

*m/z* Calculated Mass (ESI^+^): 2171.92 [M]^+^, 543.98 [M+4H]^4+^, 435.38 [M+5H]^5+^, 362,98 [M+6H]^6+^. Mass Found: 544 [M+4H]^4+^, 435 [M+5H]^5+^, 363 [M+6H]^6+^.

**Preparation of BODIPY labelled peptides**

To a solution of CK_16_ (2.3 µmol in 1 mL methanol:water (80:20)) was added maleimido-BODIPY (3.0 mg, 4.5 µmol) followed by solid NaHCO_3_ (1.8 mg, 21 µmol). The reaction was stirred for 2 h at room temperature. The solvent was then evaporated and the crude purified via preparative HPLC using acetonitrile/water/0.01% TFA to yield 0.5 mg of F4-BODIPY-CK_16_ (0.175 µmol, 8%).

The reaction was repeated with the peptide CK_16_RVRRYHWYGYTPQNVI to yield 0.7 mg of F4-BODIPY-CK_16_RVRRYHWYGYTPQNVI (0.142 µmol 6%).

**F4-BODIPY-C-K16 (P8)**

Analytical HPLC trace of purified F4-BODIPY-C-K16; R_t_ 14.4 min (5-85% B over 20 min, λ = 220 nm).


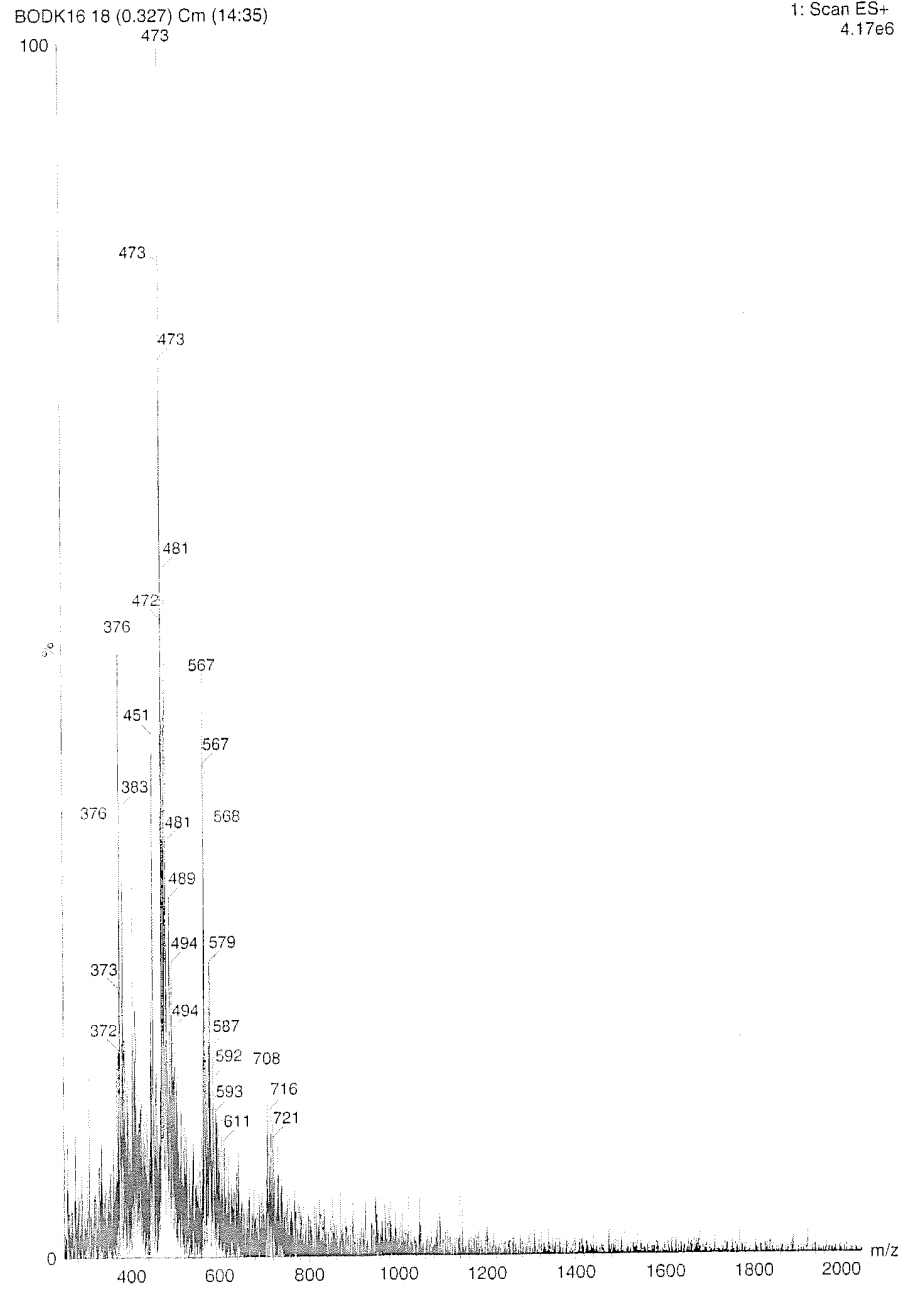


*m/z* Calculated Mass (ESI^+^): 2831.15 [M-F]^+^  708,78 [M-F+4H]^4+^, 567.23 [M-F+5H]^5+^, 472.86 [M-F+6H]^6+^. Mass Found : 708 [M-F+4H]^4+^, 567 [M-F+5H]^5+^, 473 [M-F+6H]^6+^.

**F4-BODIPY-C-K16-RVRR-** **YHWYGYTPQNVI (P9)**

Analytical HPLC trace of purified F4-BODIPY-C-K16-RVRR-YHWYGYTPQNVI; R_t_ 13.9 min (5-85% B over 20 min, λ = 214 nm).

**
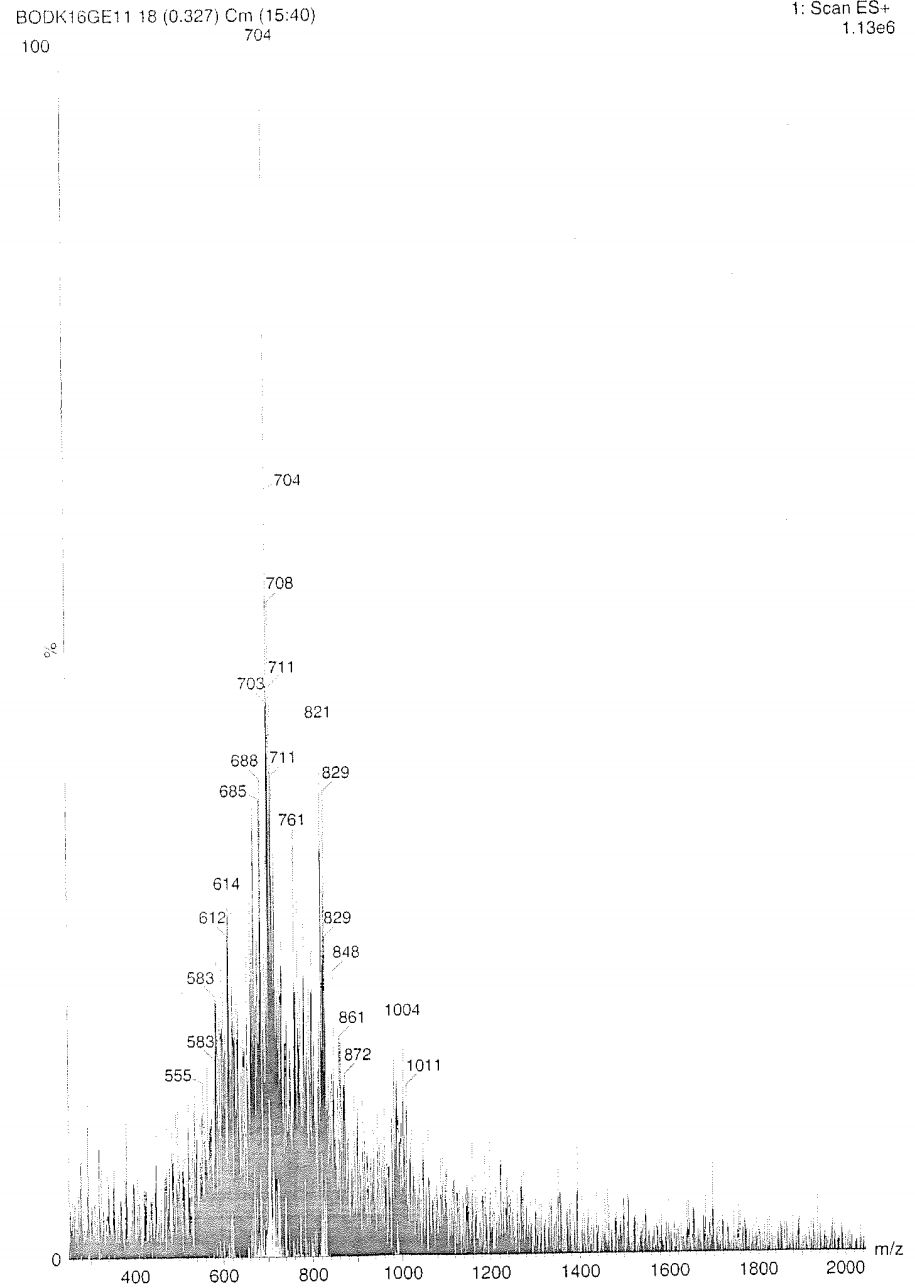
**

*m/z* Calculated Mass (ESI^+^): 4921.58 [M-F]^+^  821.16 [M-F+6H]^6+^  704.07 [M-F+7H]^7+^, 616.19 [M-F+8H]^8+^. Mass Found (ESI^+^): 821 [M-F+6H]^6+^  704 [M-F+7H]^7+^, 616 [M-F+8H]^8+^.

|  | Lipopolyplex | Lipid formulation | P1 | P2 | P3 | P4 | P5 | P6 | P7 |
| --- | --- | --- | --- | --- | --- | --- | --- | --- | --- |
| Surface | F1-(P4,P7) | F1 |  |  |  | **X** |  |  | **X** |
| targeted | F1-(P5,P7) | F1 |  |  |  |  | **X** |  | **X** |
|  | F1-(P6,P7) | F1 |  |  |  |  |  | **X** | **X** |
|  | F2-(P4,P7) | F2 |  |  |  | **X** |  |  | **X** |
|  | F2-(P5,P7) | F2 |  |  |  |  | **X** |  | **X** |
|  | F2-(P6,P7) | F2 |  |  |  |  |  | **X** | **X** |
|  | F3-(P5,P7) | F3 |  |  |  |  | **X** |  | **X** |
|  | F4-(P5,P7) | F4 |  |  |  |  | **X** |  | **X** |
|  | F5-(P5,P7) | F5 |  |  |  |  | **X** |  | **X** |
|  | F6-(P5,P7) | F6 |  |  |  |  | **X** |  | **X** |
| Bimodal | F7-(P1) | F7 | **X** |  |  |  |  |  |  |
| peptide | F7-(P2) | F7 |  | **X** |  |  |  |  |  |
|  | F7-(P3) | F7 |  |  | **X** |  |  |  |  |
|  | F7-(P7)-REF | F7 |  |  |  |  |  |  | **X** |
|  | F8-(P1) | F8 | **X** |  |  |  |  |  |  |
|  | F8-(P2) | F8 |  | **X** |  |  |  |  |  |
|  | F8-(P3) | F8 |  |  | **X** |  |  |  |  |
| Surface | F9-(P4,P7) | F9 |  |  |  | **X** |  |  | **X** |
| targeted | F9-(P5,P7) | F9 |  |  |  |  | **X** |  | **X** |
|  | F9-(P6,P7) | F9 |  |  |  |  |  | **X** | **X** |

**Table S2:** Summary of lipopolyplexes prepared for the transfection of HCC1954 cells. The names of the lipopolyplexes are made up as follows “Lipid formulation – (Peptides used)”.

**Characterisation of liposomes and lipopolyplexes**

The liposomes and lipopolyplexes were characterized using dynamic light scattering (DLS) and zeta potential measurements. Data were obtained using a Malvern Zetasizer Nano-ZS (Malvern, UK); aliquots of 20 µL were diluted to 1 mL in sterilized water and analysed in triplicate at 25 °C.

|  | Size (nm) | PDI | Zeta potential (mV) |
| --- | --- | --- | --- |
| Surface targeted liposomes |  |  |  |
| **F2-(P4)** | 133 ± 7 | 0.27 ± 0.04 | +23 ± 2.5 |
| **F2-(P5)** | 126 ± 6 | 0.33 ± 0.01 | +26 ± 2.0 |
| **F2-(P6)** | 122 ± 7 | 0.43 ± 0.04 | +23 ± 2.3 |
| Untargeted liposome |  |  |  |
| **F7** | 286 ± 6 | 0.42 ± 0.02 | +43 ± 1.5 |

**Table S3:** Biophysical characterisation of surface targeted liposomes **F2-(P4)**, **F2-(P5)**, **F2-(P6)** and of untargeted liposome **F7**.

|  | Size (nm) | PDI | Zeta potential (mV) |
| --- | --- | --- | --- |
| **F2-(P4,P7)** | 258 ± 89 | 0.32 ± 0.04 | +33 ± 1 |
| **F2-(P5,P7)** | 447 ± 60 | 0.46 ± 0.04 | +15 ± 1 |
| **F2-(P6,P7)** | 203 ± 12 | 0.39 ± 00.2 | +26 ± 2 |
| **F7-(P1)** | 294 ± 23 | 0.14 ± 0.03 | +33 ± 5 |
| **F7-(P2)** | 222 ± 5 | 0.29 ± 0.01 | +15 ± 1 |
| **F7-(P3)** | 284± 45 | 0.25 ± 0.02 | +21 ± 1 |
| **F7-(P7)** | 383 ± 98 | 0.41 ± 0.05 | +25 ± 1 |

**Table S4:** Biophysical characterisation of lipopolyplexes after complexation with pDNA

**Plasmid Map**

**Labelling of plasmid DNA**

The pDNA solution was diluted to a concentration of 1 mg/mL and labelled with a functionalised fluorescein fluorophore using a commercially available labelling kit (Label IT® Tracker™ Intracellular Nucleic Acid Localization Kit, Fluorescein) No alterations to the standard protocol were made.

**Fluorescence quenching**

**Figure S1:** Fluorescence quenching of free and liposomal fluorescein (5(6)-Carboxyfluorescein 100 µM, DPPC 1 mM in HEPES 20 mM, pH 7.4, λ_exc_ = 466, λ_em_ = 516 ).


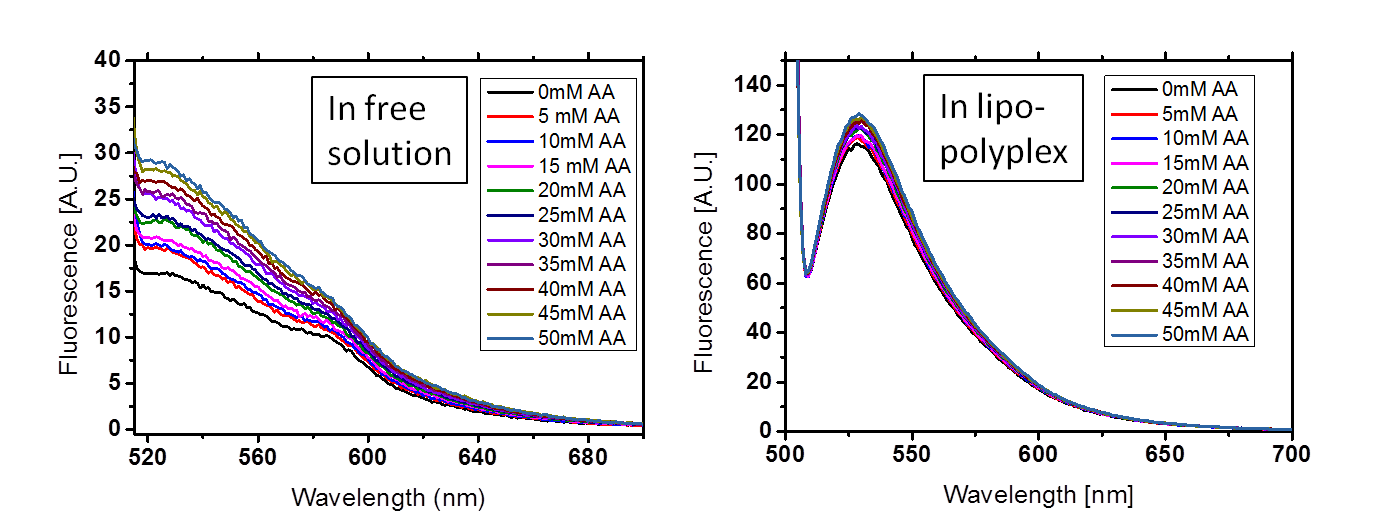


**Figure S2:** Fluorescein labeled pDNA emission in free solution and in lipopolyplex **F7-(P1)**, overall lipid concentration = 200 µM, peptide = 10 µM, Luciferase plasmid DNA 0.02 µg/µL)), λ_exc_ = 491 upon addition of 0-50 mM acrylamide.

**References**

1. Galangau, O.; Dumas-Verdes, C.; Meallet-Renault, R.; Clavier, G. *Org. Biomol. Chem.* **2010**, *8*, 4546.
2. Hurley, C. A.; Wong, J. B.; Hailes, H. C.; Tabor, A. B. *J. Org. Chem.* **2004**, *69*, 980.
3. Mitchell, N.; Kalber, T. L.; Cooper, M. S.; Sunassee, K.; Chalker, S. L.; Shaw, K. P.; Ordidge, K. L.; Badar, A.; Janes, S. M.; Blower, P. J.; Lythgoe, M. F.; Hailes, H. C.; Tabor, A. B. *Biomaterials* **2013**, *34*, 1179.
4. Weitsman, G.; Mitchell, N. J.; Evans, R.; Cheung, A.; Kalber, T. L.; Bofinger, R.; Fruhwirth, G. O.; Keppler, M.; Wright, Z. V. F.; Barber, P. R.; Gordon, P.; de Koning, T.; Wulaningsih, W.; Sander, K.; Vojnovic, B.; Ameer-Beg, S.; Lythgoe, M.; Arnold, J. N.; Arstad, E.; Festy, F.; Hailes, H. C.; Tabor, A. B.; Ng, T. *Oncogene* **2017**, *36*, 3618.
